# Supplementary material for: Analysis of the 2007–2018 National Health Interview Survey (NHIS): Examining Neurological Complications among Children with Sickle Cell Disease in the United States
Source: Int J Environ Res Public Health. 2023 Jun 15;20(12):6137. doi: 10.3390/ijerph20126137 (PMC10298081; doi:10.3390/ijerph20126137)
Supplement: Supplementary file 1 [file ijerph-20-06137-s001.zip › Supplement Figures_1A-5N_SCD_Neuro_06122023.pdf]

Supplemental Figures

S1 Figure Predictive margins of interaction between sickle cell disease status and insurance coverage on **ADD/ADHD** with 95% Confidence Interval

S2a Figure Predictive margins of interaction between sickle cell disease status and age on **Learning Disability** with 95% Confidence Interval

S2b Figure Predictive margins of interaction between sickle cell disease status and insurance coverage on **Learning Disability** with 95% Confidence Interval

S3a Figure Predictive margins of interaction between sickle cell disease status and sex on **Took prescription medication for at least three months** with 95% Confidence Interval

S3b Figure Predictive margins of interaction between sickle cell disease status and age on **Took prescription medication for at least three months** with 95% Confidence Interval

S4a Figure Predictive margins of interaction between sickle cell disease status and insurance coverage on **Saw a therapist** with 95% Confidence Interval

S4b Figure Predictive margins of interaction between sickle cell disease status and household income (% federal poverty level) coverage on **Saw an optometrist, ophthalmologist, or eye doctor** with 95% Confidence Interval

S5a Figure. Predictive margins of interaction between sickle cell disease status and maternal education on **Multiple visits to emergency room: none with 95% Confidence Interval**

S5b Figure Predictive margins of interaction between sickle cell disease status and insurance coverage on **Multiple visits to emergency room: none** with 95% Confidence Interval

S5c Figure Predictive margins of interaction between sickle cell disease status and maternal education on **Multiple visits to emergency room: 1** with 95% Confidence Interval

S5d Figure Predictive margins of interaction between sickle cell disease status and age on **Multiple visits to emergency room: 2-3** with 95% Confidence Interval

S5e Figure Predictive margins of interaction between sickle cell disease status and maternal education on **Multiple visits to emergency room: 4-5** with 95% Confidence Interval

S5f Figure Predictive margins of interaction between sickle cell disease status and age on Multiple visits to emergency room: 4-5 with 95% Confidence Interval

S5g Figure Predictive margins of interaction between sickle cell disease status and household income (% federal poverty level) on **Multiple visits to emergency room: 4-5** with 95% Confidence Interval

S5h Figure Predictive margins of interaction between sickle cell disease status and private insurance on **Multiple visits to emergency room: 4-5** with 95% Confidence Interval

S5i Figure Predictive margins of interaction between sickle cell disease status and age on **Multiple visits to emergency room: 6-16+** with 95% Confidence Interval

S5j Figure Predictive margins of interaction between sickle cell disease status and private insurance on **Multiple visits to emergency room: 6-16+** with 95% Confidence Interval

S5k Figure Predictive margins of interaction between sickle cell disease status and sex on **Had surgery or another surgical procedure** with 95% Confidence Interval

S5l Figure Predictive margins of interaction between sickle cell disease status and household income (% federal poverty level) on **Had surgery or another surgical procedure** with 95% Confidence Interval

S5m Figure Predictive margins of interaction between sickle cell disease status and public insurance on **Had surgery or another surgical procedure** with 95% Confidence Interval

S5n Figure Predictive margins of interaction between sickle cell disease status and private insurance on **Had surgery or another surgical procedure** with 95% Confidence Interval

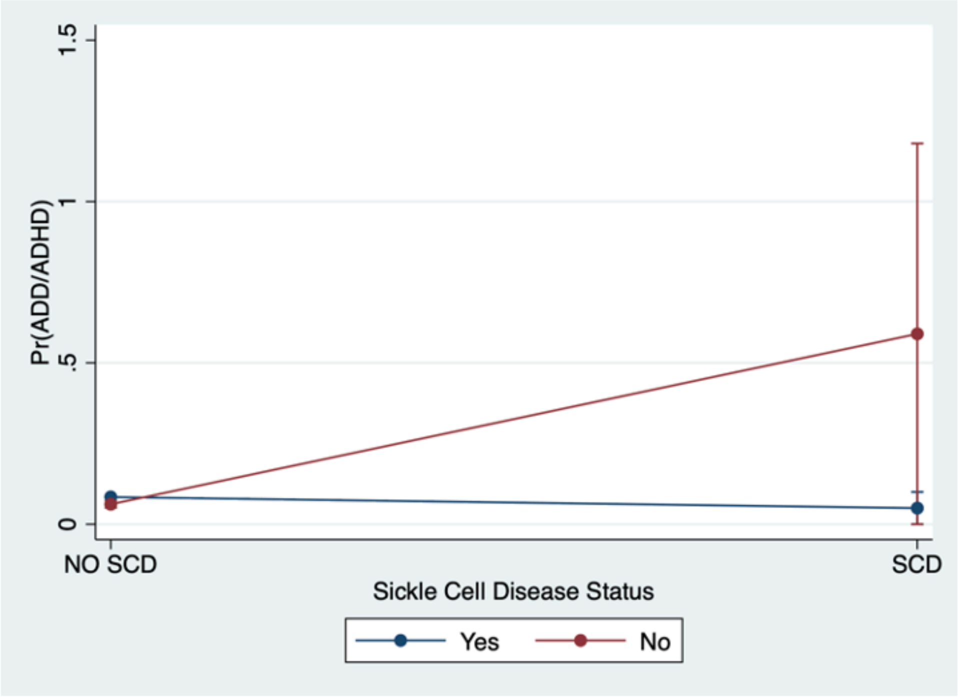

Supplemental Figure 1. Predictive margins of interaction between sickle cell disease status and insurance coverage on **ADD/ADHD** with 95% Confidence Interval

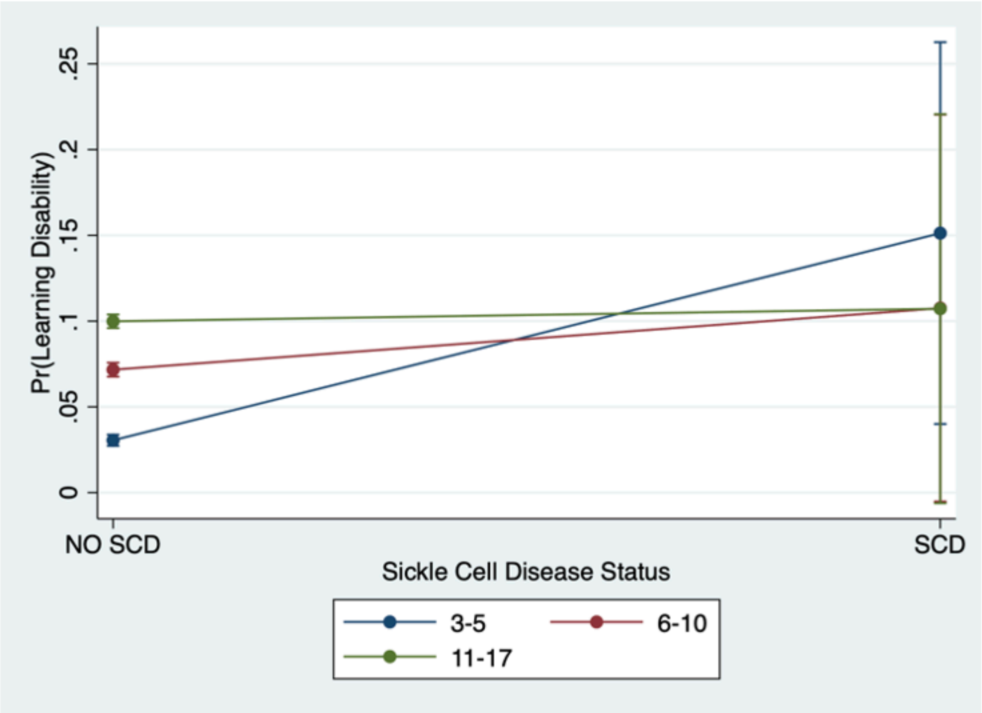

Supplemental Figure 2a. Predictive margins of interaction between sickle cell disease status and age on **Learning Disability** with 95% Confidence Interval

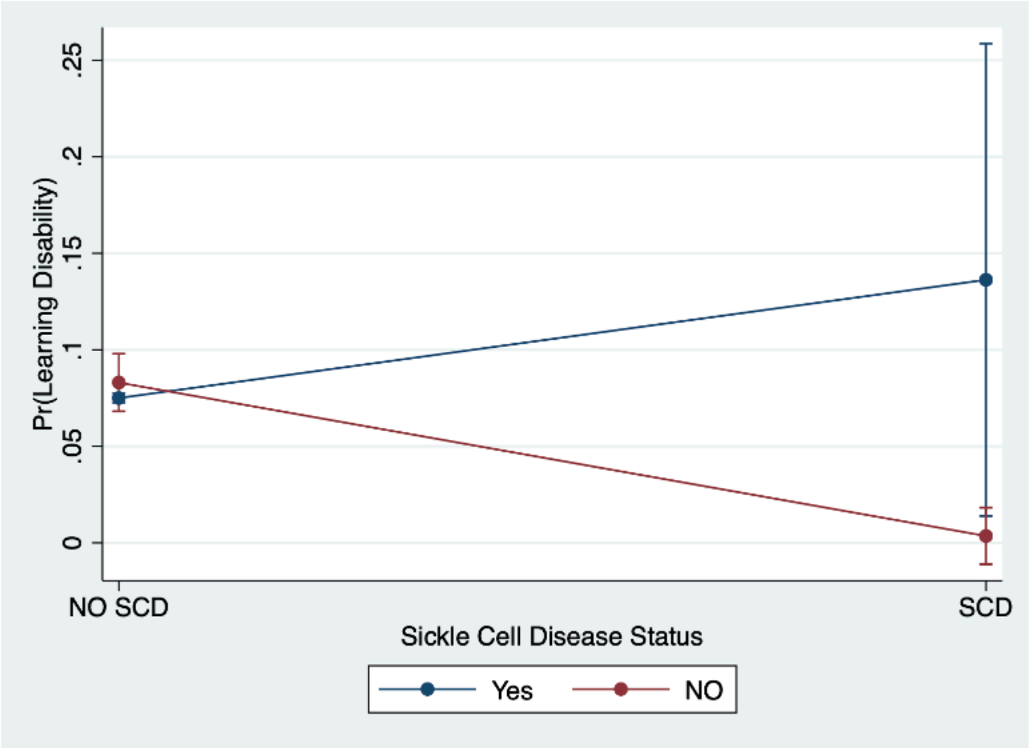

Supplemental Figure 2b. Predictive margins of interaction between sickle cell disease status and insurance coverage on **Learning Disability** with 95% Confidence Interval

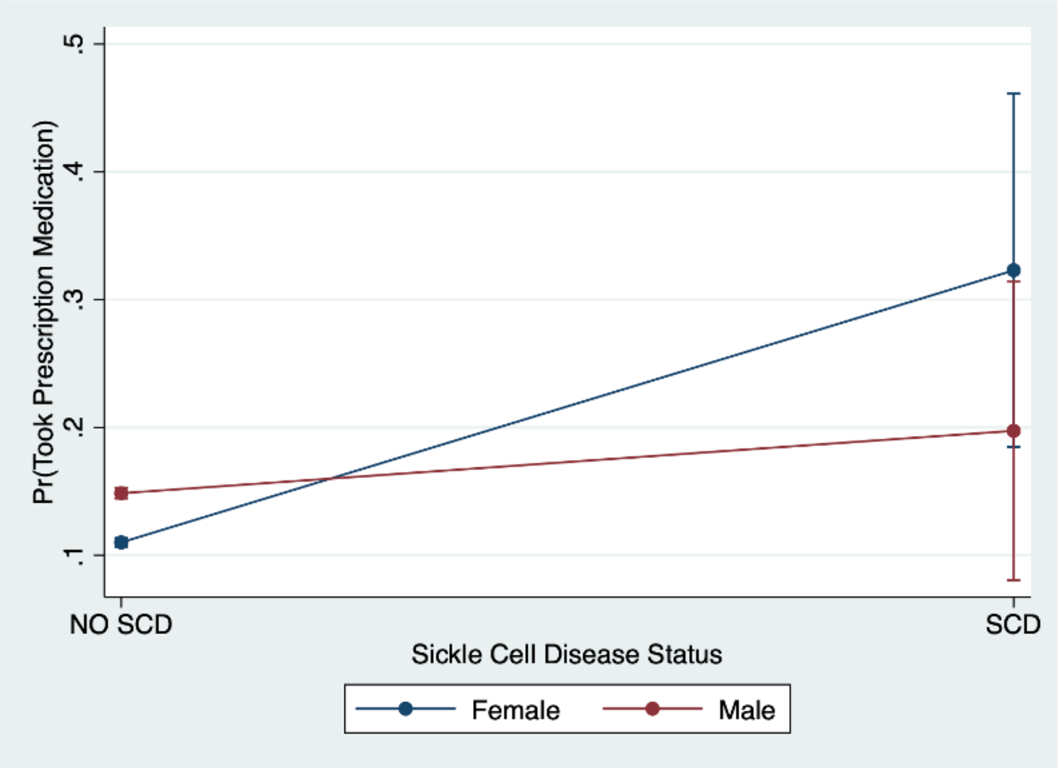

Supplemental Figure 3a. Predictive margins of interaction between sickle cell disease status and sex on **Took prescription medication for at least three months** with 95% Confidence Interval

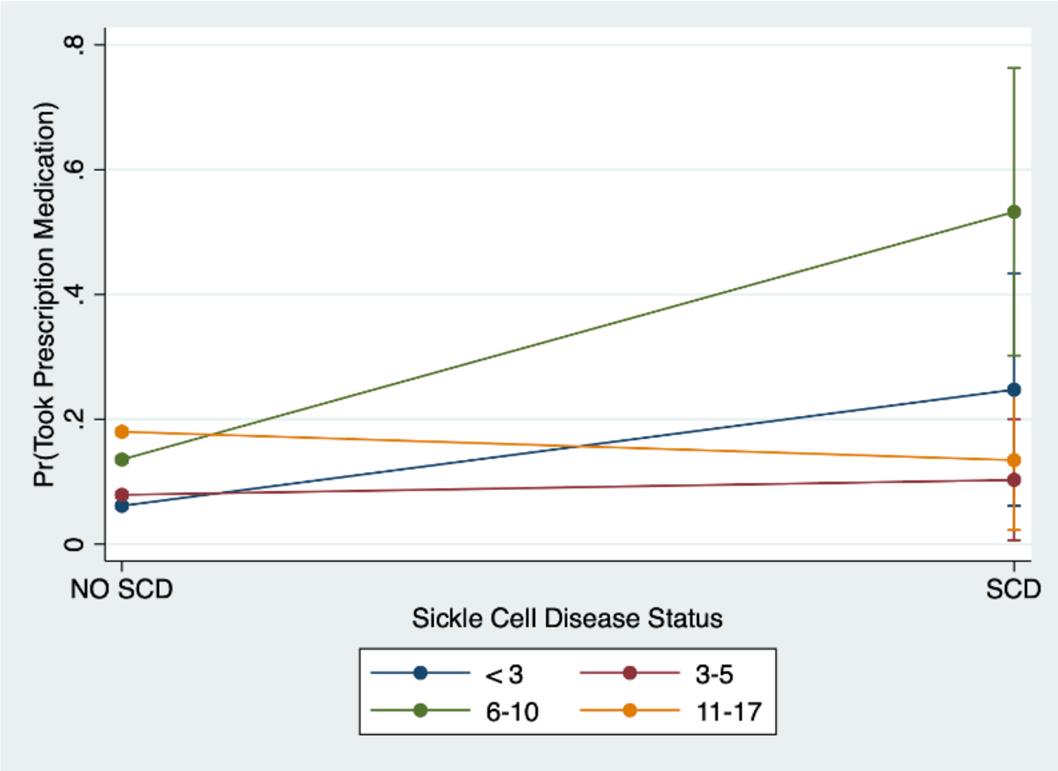

Supplemental Figure 3b. Predictive margins of interaction between sickle cell disease status and age on **Took prescription medication for at least three months** with 95% Confidence Interval

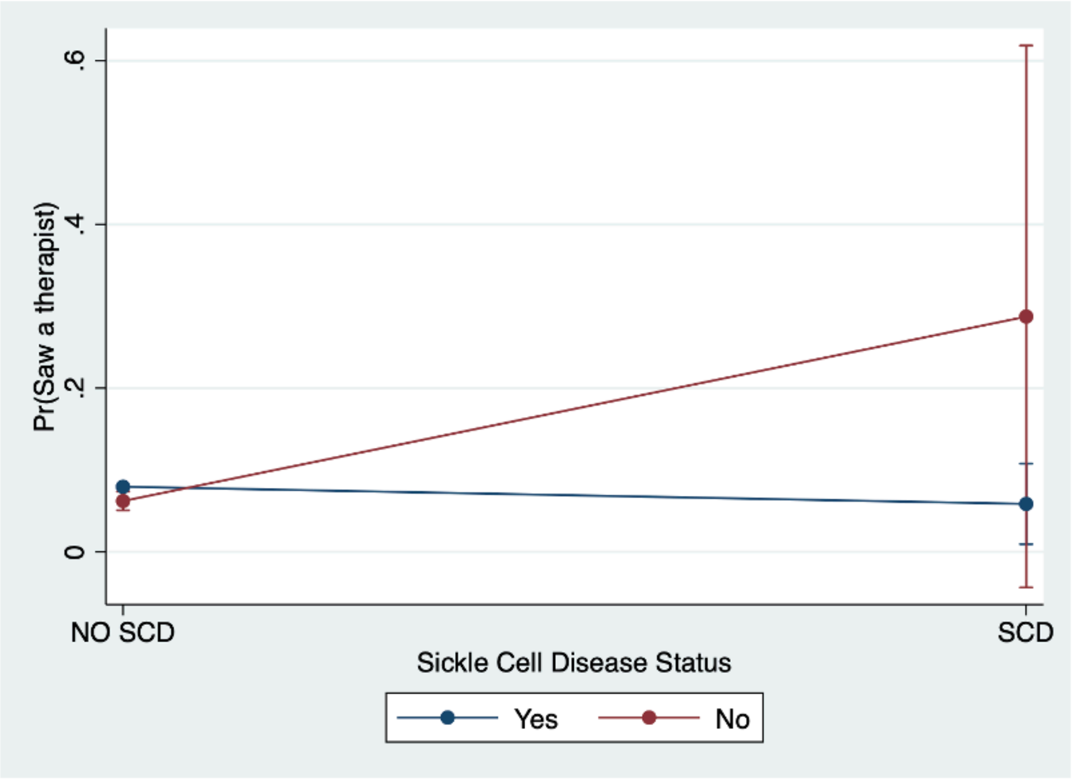

Supplemental Figure 4a. Predictive margins of interaction between sickle cell disease status and insurance coverage on **Saw a therapist** with 95% Confidence Interval

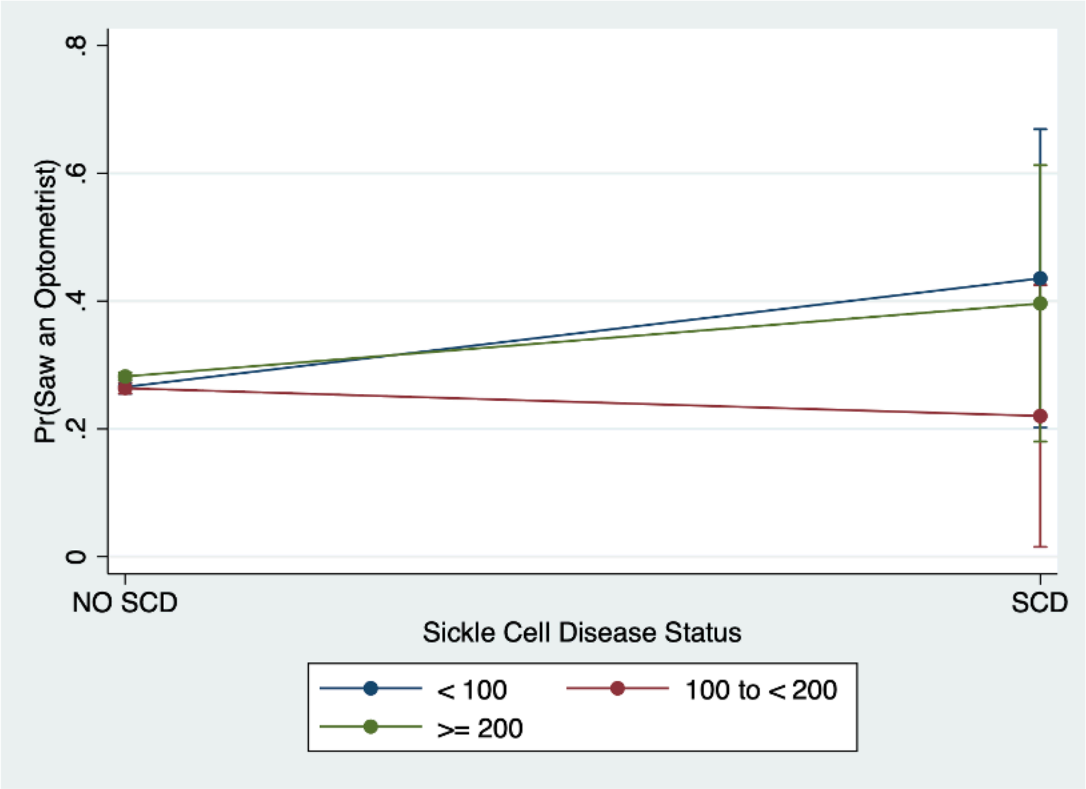

Supplemental Figure 4b. Predictive margins of interaction between sickle cell disease status and household income (% federal poverty level) coverage on **Saw an optometrist, ophthalmologist, or eye doctor** with 95% Confidence Interval

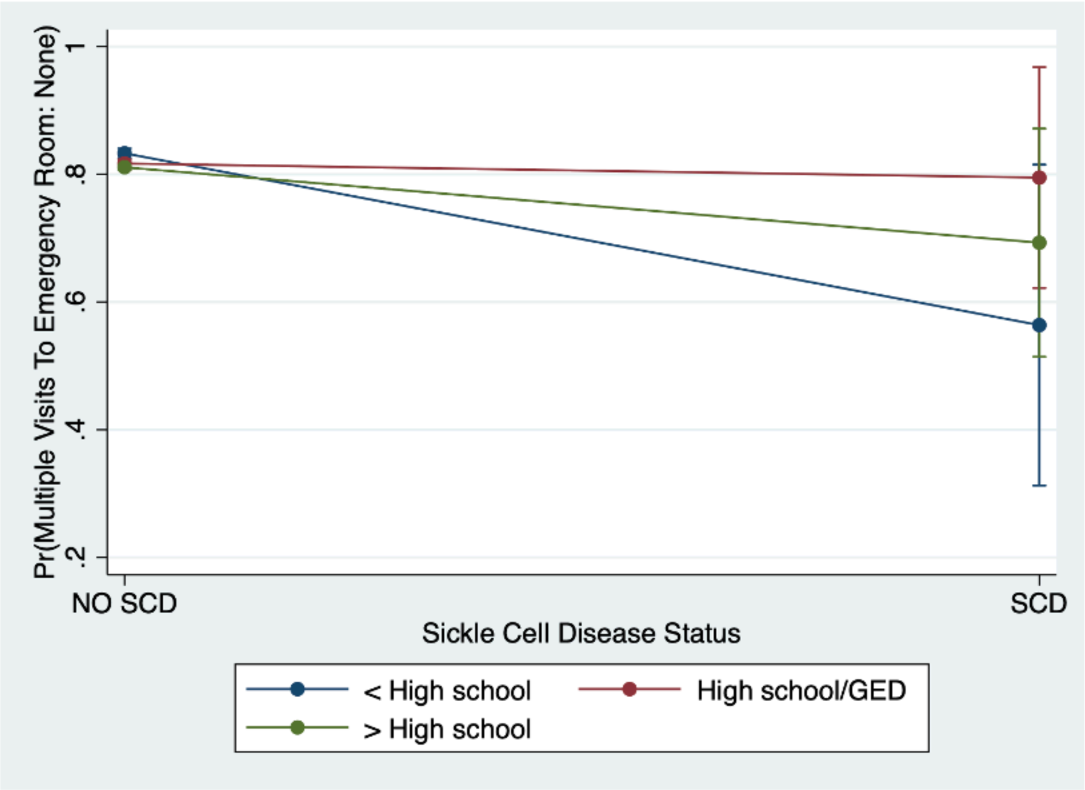

Supplemental Figure 5a. Predictive margins of interaction between sickle cell disease status and maternal education on **Multiple visits to emergency room: none with 95% Confidence Interval**

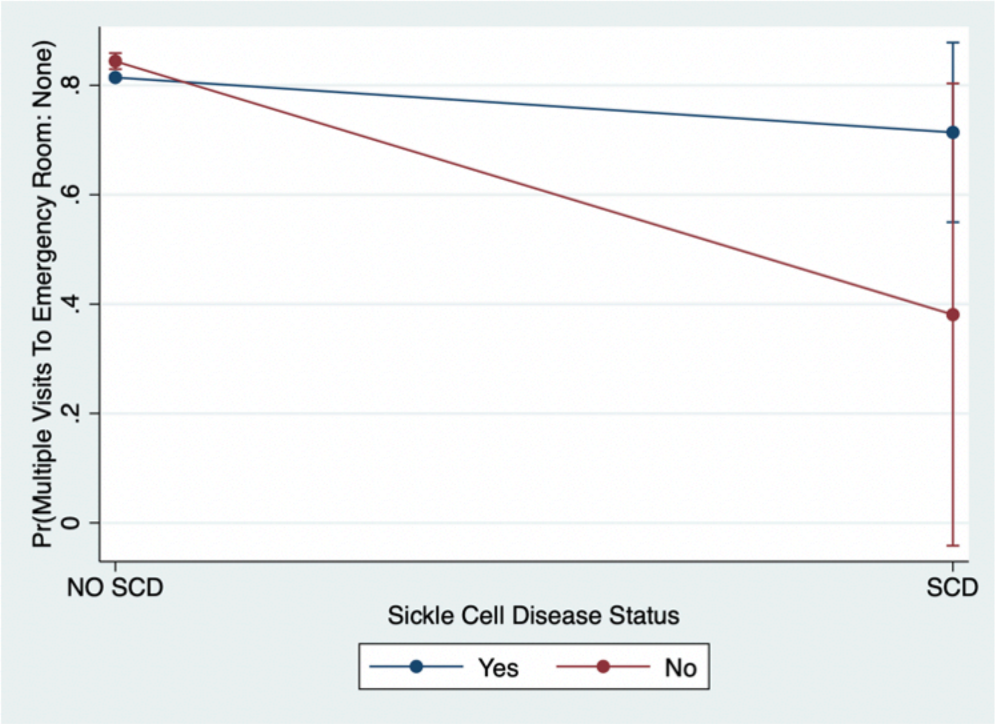

Supplemental Figure 5b. Predictive margins of interaction between sickle cell disease status and insurance coverage on **Multiple visits to emergency room: none** with 95% Confidence Interval

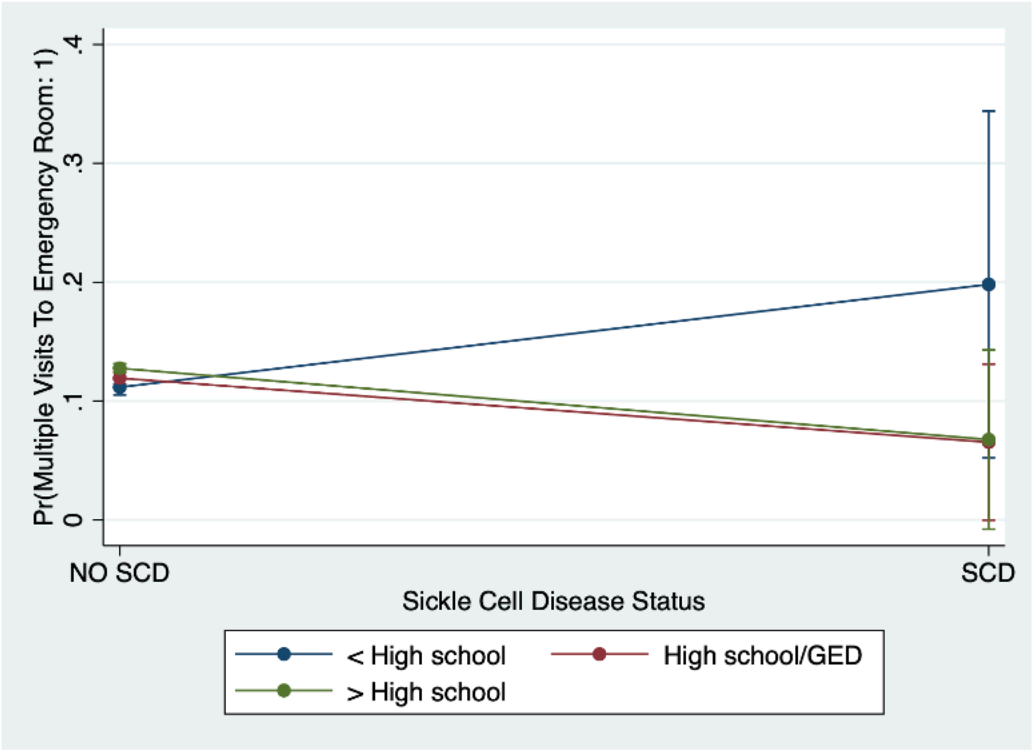

Supplemental Figure 5c. Predictive margins of interaction between sickle cell disease status and maternal education on **Multiple visits to emergency room: 1** with 95% Confidence Interval

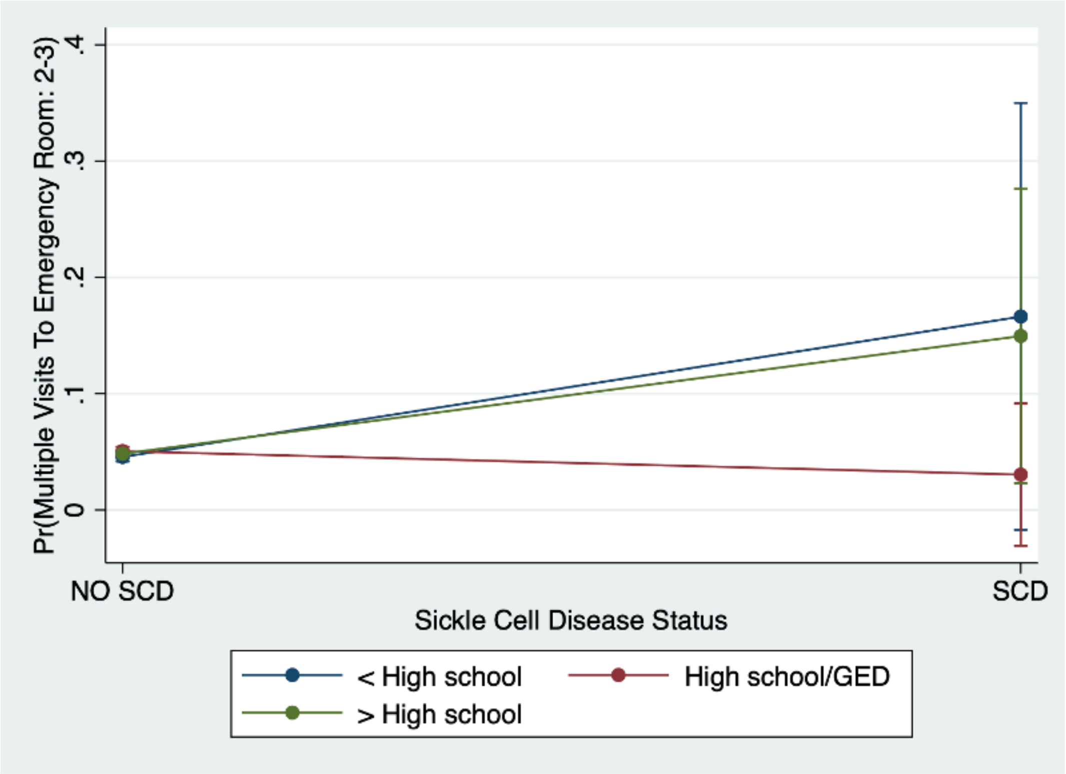

Supplemental Figure 5d. Predictive margins of interaction between sickle cell disease status and age on **Multiple visits to emergency room: 2-3** with 95% Confidence Interval

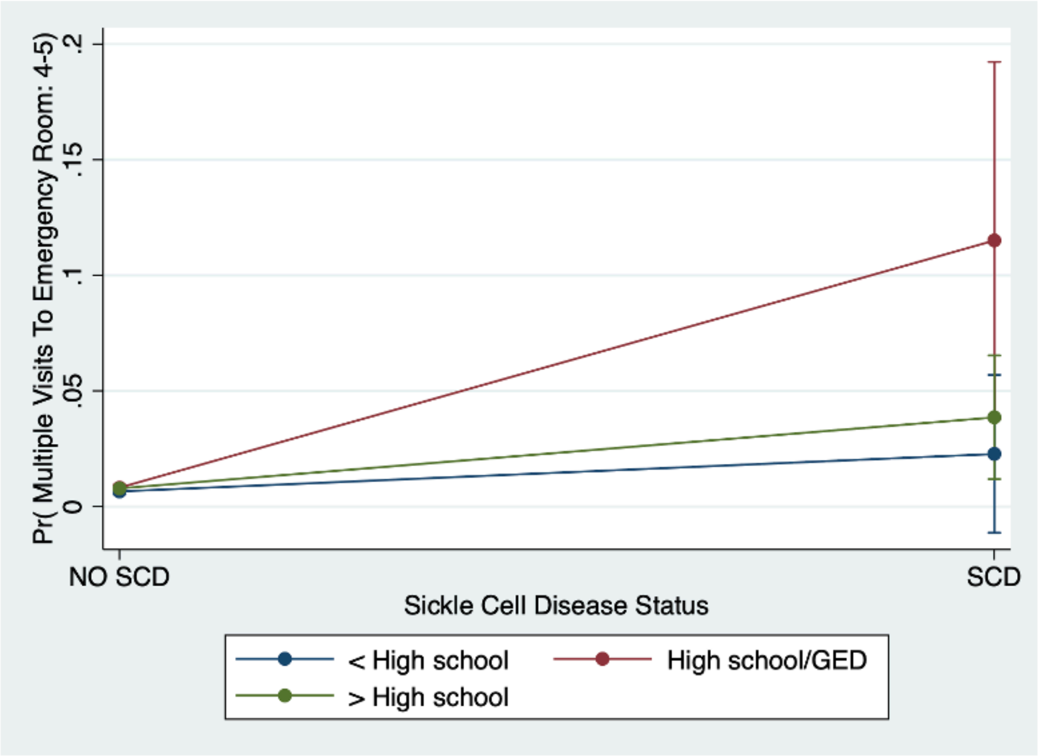

Supplemental Figure 5e. Predictive margins of interaction between sickle cell disease status and maternal education on **Multiple visits to emergency room: 4-5** with 95% Confidence Interval

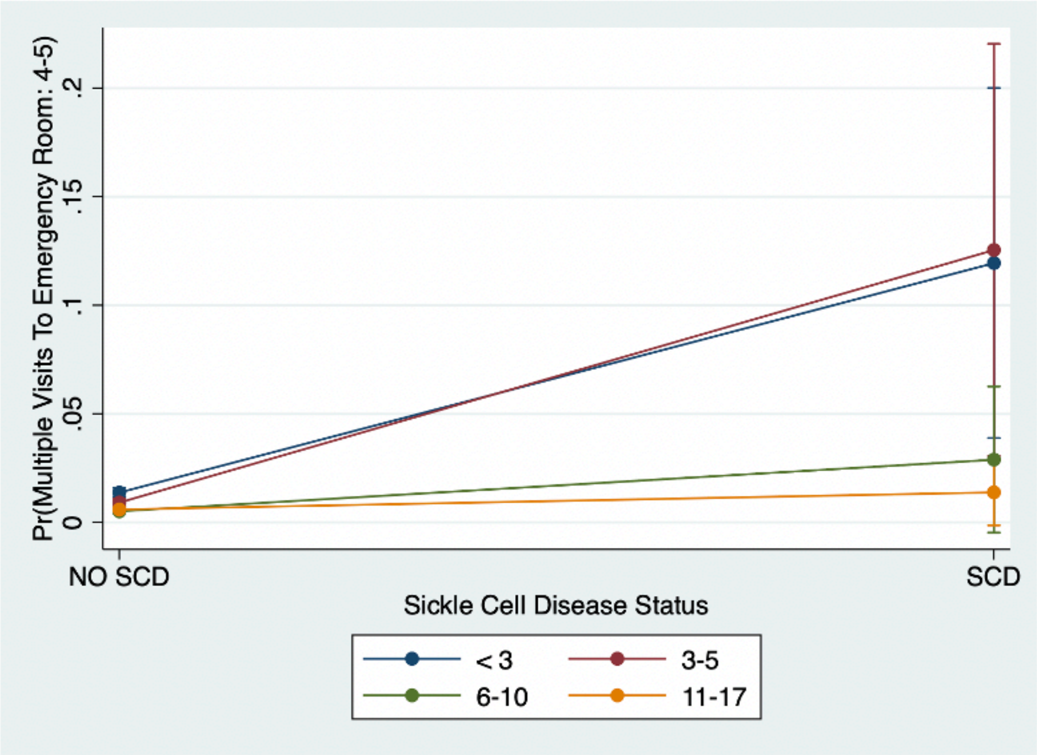

Supplemental Figure 5f. Predictive margins of interaction between sickle cell disease status and age on Multiple visits to emergency room: 4-5 with 95% Confidence Interval

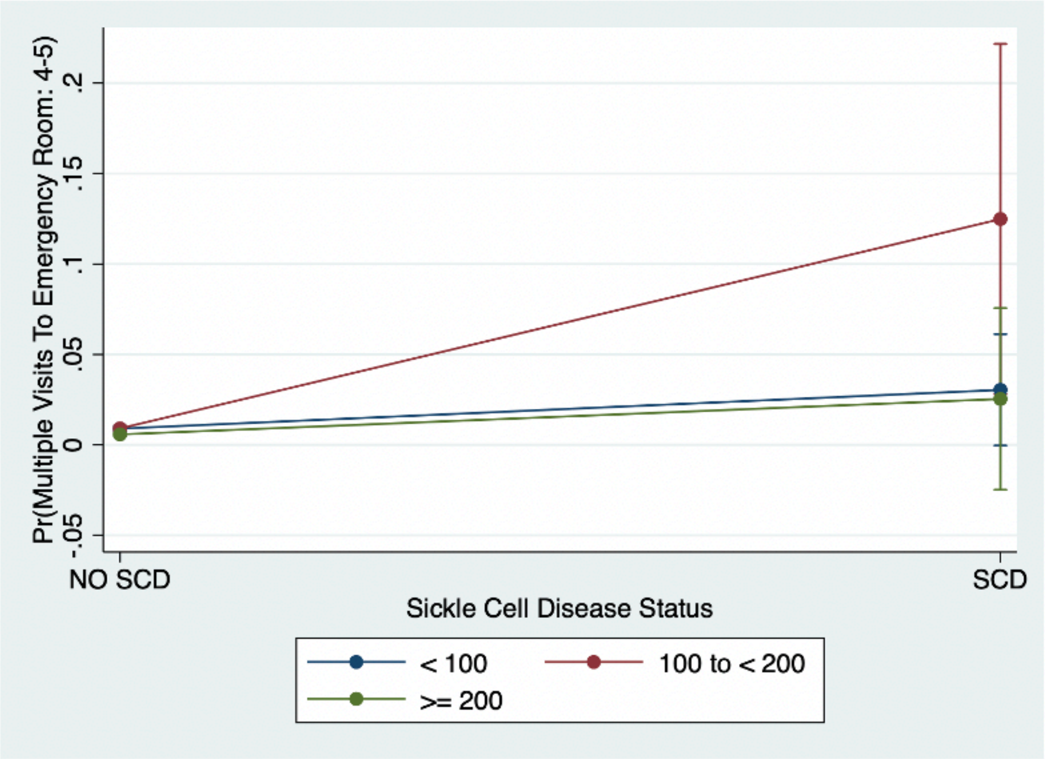

Supplemental Figure 5g. Predictive margins of interaction between sickle cell disease status and household income (% federal poverty level) on **Multiple visits to emergency room: 4-5** with 95% Confidence Interval

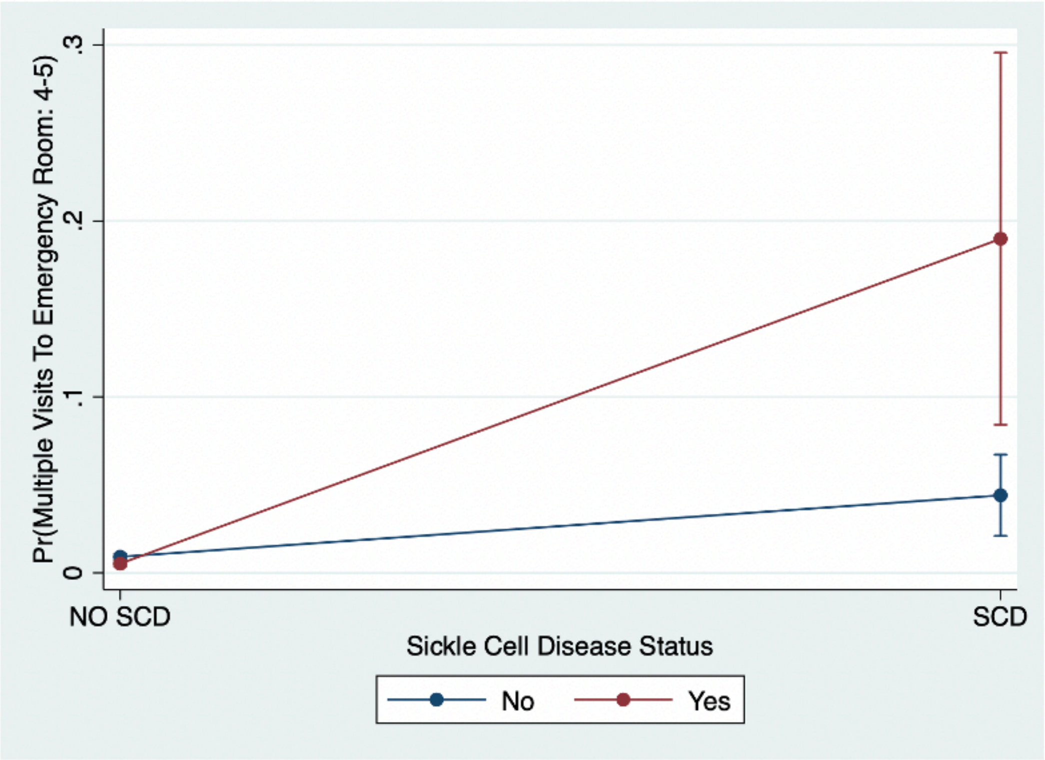

Supplemental Figure 5h. Predictive margins of interaction between sickle cell disease status and private insurance on **Multiple visits to emergency room: 4-5** with 95% Confidence Interval

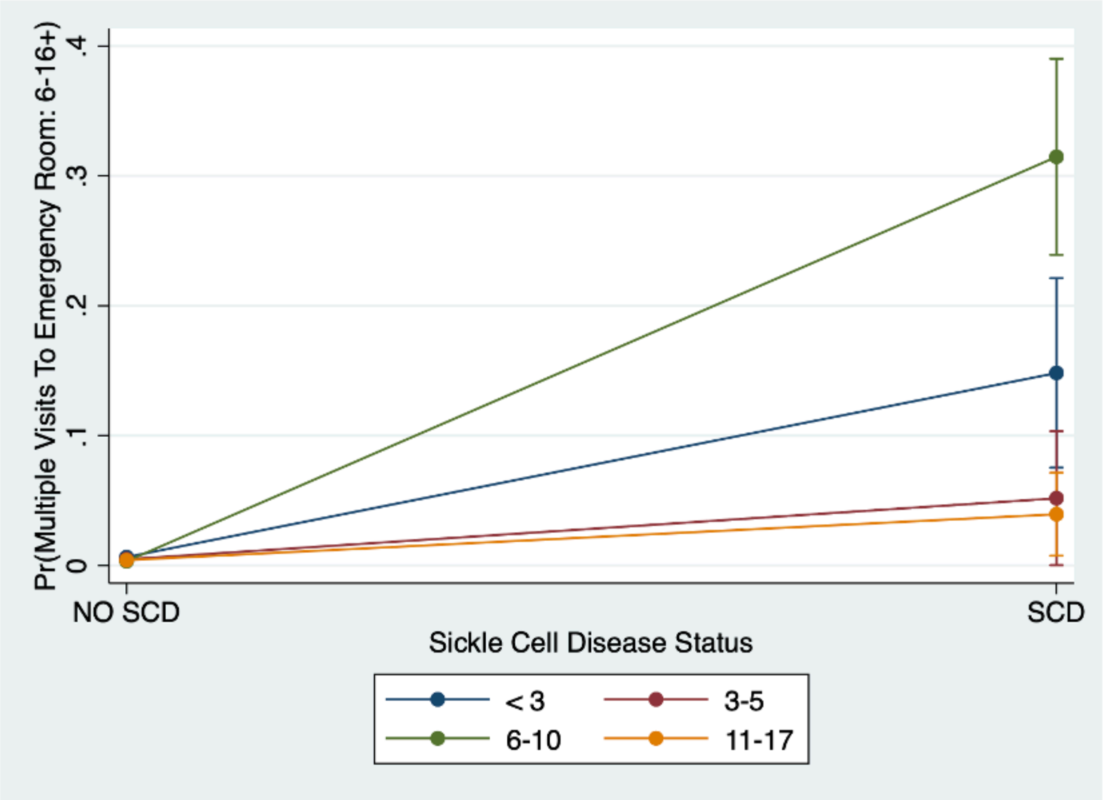

Supplemental Figure 5i. Predictive margins of interaction between sickle cell disease status and age on **Multiple visits to emergency room: 6-16+** with 95% Confidence Interval

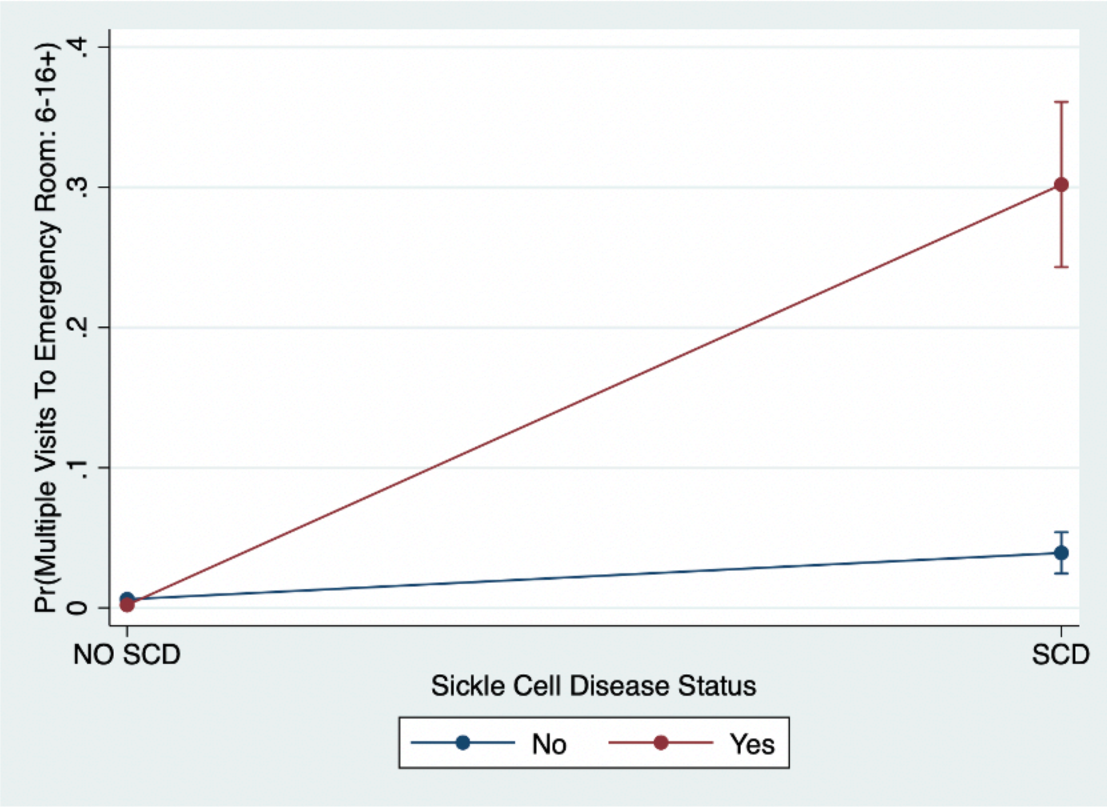

Supplemental Figure 5j. Predictive margins of interaction between sickle cell disease status and private insurance on **Multiple visits to emergency room: 6-16+** with 95% Confidence Interval

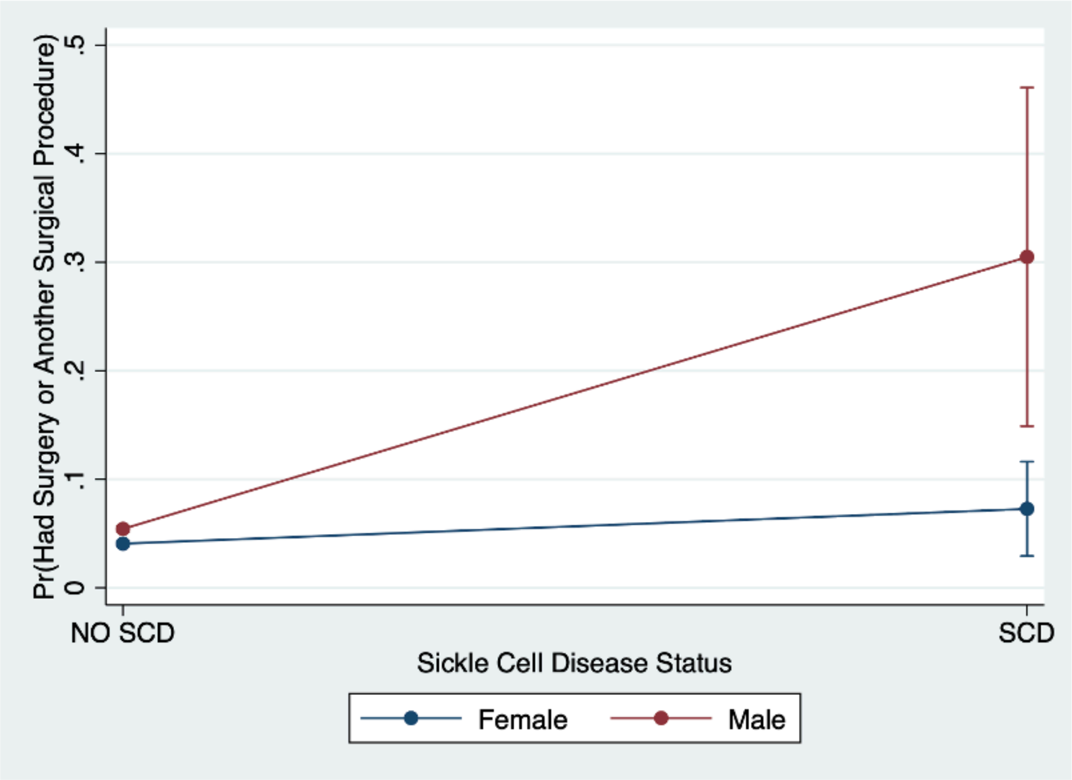

Supplemental Figure 5k. Predictive margins of interaction between sickle cell disease status and sex on **Had surgery or another surgical procedure** with 95% Confidence Interval

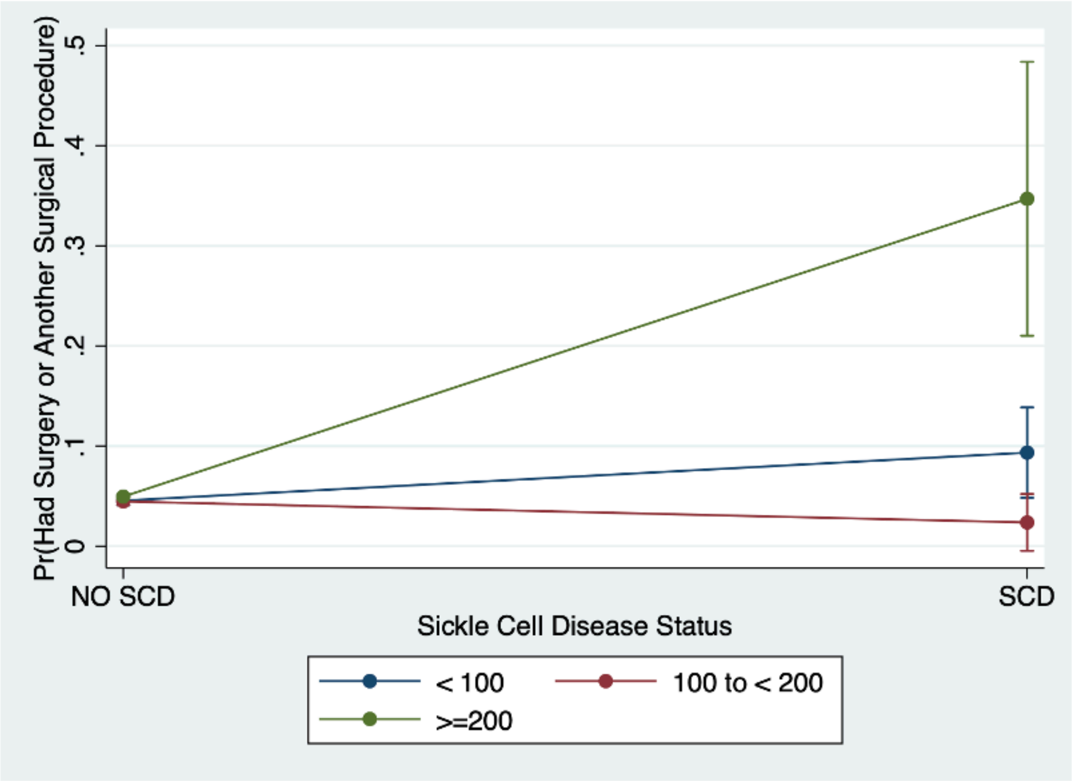

Supplemental Figure 5l. Predictive margins of interaction between sickle cell disease status and household income (% federal poverty level) on **Had surgery or another surgical procedure** with 95% Confidence Interval

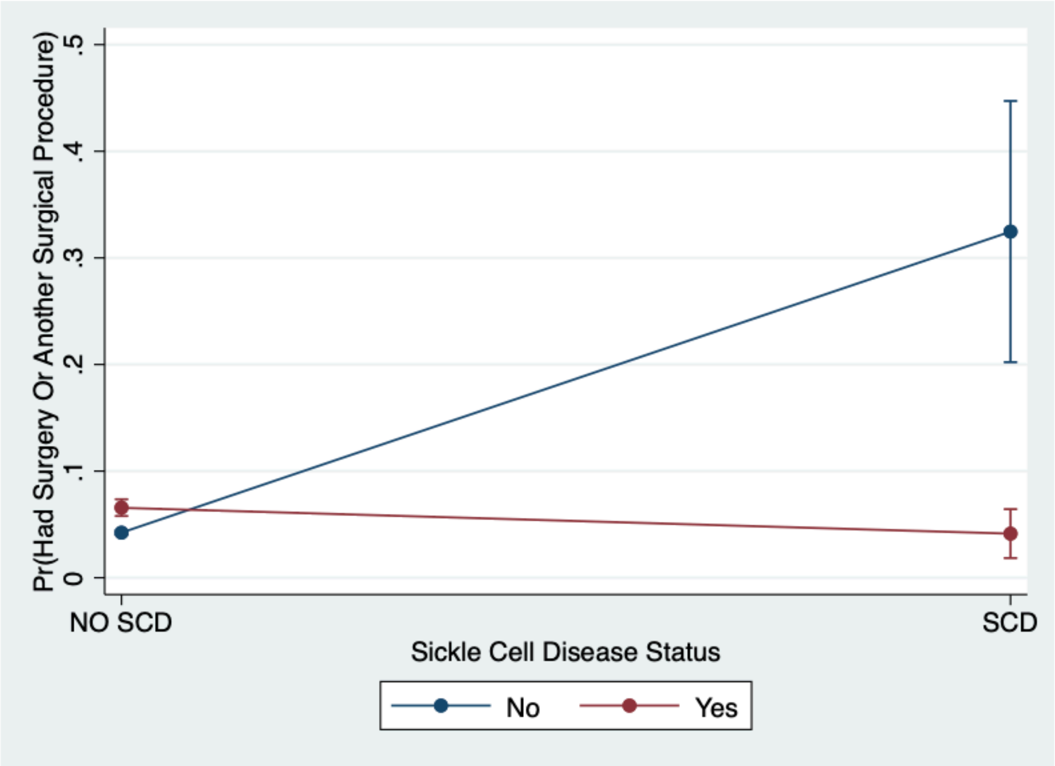

Supplemental Figure 5m. . Predictive margins of interaction between sickle cell disease status and public insurance on **Had surgery or another surgical procedure** with 95% Confidence Interval

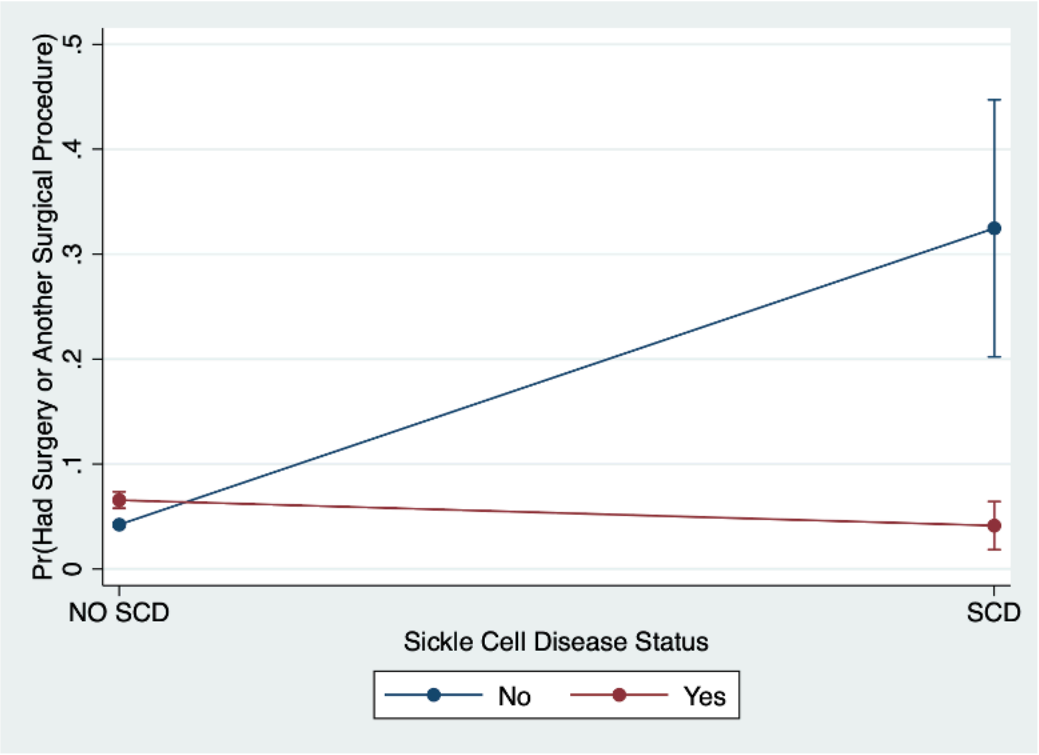

Supplemental Figure 5n. Predictive margins of interaction between sickle cell disease status and private insurance on **Had surgery or another surgical procedure** with 95% Confidence Interval
