# Supplementary material for: Analysis of the 2007–2018 National Health Interview Survey (NHIS): Examining Neurological Complications among Children with Sickle Cell Disease in the United States
Source: Int J Environ Res Public Health. 2023 Jun 15;20(12):6137. doi: 10.3390/ijerph20126137 (PMC10298081; doi:10.3390/ijerph20126137)
Supplement: Supplementary file 1 [file ijerph-20-06137-s001.zip › Supplemental Tables 1 2 3_revised_06122023.pdf]

Supplemental Tables.
Supplemental Table S1. Demographic characteristics of NHIS children by Sickle Cell Disease (SCD) status
Supplemental Table S2. Demographic characteristics of Black children with and without SCD
Supplemental Table S3. Interaction Effects Between Sickle Cell Disease Status and SES in Multivariable Regression Models

Supplemental Table S1: Demographic characteristics of NHIS children by Sickle Cell Disease (SCD) status

	**Children with SCD (n=215)**		**Children without SCD (n=133,266)**		**P value**
**Year of Survey**	Unweighted n	Weighted %	Unweighted n	Weighted %	
2007-2010	79	36.23	40,566	33.46	0.701
2011-2014	85	33.70	52,254	33.33	
2015-2018	51	30.07	40,446	33.21	
**Child Gender**					
Male	110	48.23	68,593	51.07	0.523
Female	105	51.77	64,673	48.93	
**Child Age (years)**					
<3	42	16.30	23,052	16.23	0.242
3-5	45	23.75	21,571	16.99	
6-10	49	22.47	34,053	27.71	
11-14	37	20.42	29,077	22.15	
15-17	42	17.06	25,513	16.92	
**Race**					
White	16	7.66	85,965	68.83	**<0.001**
Black/African American	170	81.81	21,395	15.34	
Indian(American Indian)	1	0.57	1,847	1.34	
Asian	2	1.22	5,458	3.12	
Other race	21	6.41	16,705	10.13	

Multiple race	5	2.33	1,896	1.25	
**Maternal Education**					
< High school	40	19.02	19,310	14.81	**0.029**
High School/GED	54	28.58	27,421	21.68	
> High school	99	52.41	75,560	63.51	
Missing	22		10,975		
**Household income (% federal poverty level)**					
<100	97	53.81	23,303	21.00	**<0.001**
100 to <200	42	22.51	26,787	22.94	
≥ 200	42	23.68	64,984	56.05	
Missing	34		18,192		
**Insurance**					
Medicaid/SCHIP*	111	53.85	32,653	25.84	**<0.001**
Private insurance	51	27.63	62,976	52.12	
Other insurance	18	7.42	10,346	7.92	
No insurance	17	7.77	10,405	7.43	
Multiple Insurance	8	3.33	8,043	6.69	
Missing	10		8,843		
**Region**					
Northeast	30	14.56	20,949	16.27	**<0.001**
Midwest	50	30.40	27,091	22.82	
South	116	49.07	48,356	36.87	
West	19	5.97	36,870	24.04	

*SCHIP: State Child Health Insurance Program

Supplemental Table S2: Demographic characteristics of Black children with and without SCD

	Black Children with SCD (n=170)		Black Children without SCD (n=21,395)		P value
**Year of Survey**	Unweighted n	Weighted %	Unweighted n	Weighted %	
2007-2010	68	40.66	7,175	33.88	0.183
2011-2014	66	34.12	8,630	32.96	
2015-2018	36	25.21	5,590	33.16	
**Child Gender**					
Male	86	48.71	10,985	50.47	0.719
Female	84	51.29	10,410	49.53	
**Child Age (years)**					
<3	31	14.37	3,459	15.99	0.311
3-5	33	23.54	3,541	17.55	
6-10	38	20.99	5,429	27.72	
11-14	32	22.36	4,794	22.04	
15-17	36	18.74	4,172	16.70	
**Maternal Education**					
< High school	25	15.94	2,519	14.26	0.5593
High School/GED	44	30.28	5,057	26.79	
> High school	83	53.77	11,243	58.94	
Missing	18		2,576		
**Household income (% federal poverty level)**					
<100	78	55.08	5,967	36.21	**0.001**
100 to <200	32	24.72	4,998	26.89	
≥ 200	30	20.20	7,216	36.90	
Missing	30		3,214		
**Insurance**					
Medicaid/SCHIP*	91	54.84	9,313	44.51	0.155

Private insurance	42	26.86	7,147	34.33	
Other insurance	14	7.50	1,840	9.65	
No insurance	12	8.09	1,375	6.29	
Multiple Insurance	4	2.69	1,003	5.23	
Missing	7		1,717		
**Region**					
Northeast	23	15.54	3,422	16.07	**<0.001**
Midwest	44	33.89	3,747	19.07	
South	94	48.67	12,092	55.95	
West	9	1.91	2,134	8.91	

*SCHIP: State Child Health Insurance Program

Supplemental Table S3. Interaction Effects Between Sickle Cell Disease Status and SES in Multivariable Regression Models

Developmental Conditions							
Outcome	Interactions (SCD*SES)	Factor Levels	Odds Ratio	Linearized Std. Err.	t	P>|t|	[95%Conf. Interval]
**ADD/ADHD**	Gender	Female	Reference	Reference	Reference	Reference	Reference
		Male	4.51	4.69	1.45	0.148	(0.58,34.76)
	Age	<3	Reference	Reference	Reference	Reference	Reference
		3-5	6.25	6.37	1.80	0.073	(0.84,46.18)
		6-10	1.45	1.63	0.33	0.742	(0.16,13.18)
		11-17	-	-	-	-	-
	Maternal Education	< High school	Reference	Reference	Reference	Reference	Reference
		High school/GED	0.61	0.54	-0.56	0.573	(0.11,3.44)
		> High school	0.35	0.37	-1.00	0.317	(0.04,2.75)
	Region	Northeast	Reference	Reference	Reference	Reference	Reference
		Midwest	13.37	37.92	0.91	0.361	(0.05,3502.09)
		South	4.20	11.65	0.52	0.605	(0.02,972.76)
		West	88.55	265.24	1.50	0.135	(0.25,31648.99)
	Household Income (% federal poverty level)	<100	Reference	Reference	Reference	Reference	Reference
		100 to < 200	0.19	0.41	-0.78	0.437	(0.00,12.14)
		≥ 200	0.24	0.63	-0.54	0.590	(0.00,45.34)
	Race	Other	Reference	Reference	Reference	Reference	Reference
		Black	0.99	1.25	-0.01	0.995	(0.26,6099.395)
	Public Insurance	No	Reference	Reference	Reference	Reference	Reference
		Yes	39.61	101.65	1.43	0.152	(0.26,6099.40)
	Private Insurance	No	Reference	Reference	Reference	Reference	Reference
		Yes	103.21	471.10	1.02	0.310	(0.01,802748.2)
	Other Insurance	No	Reference	Reference	Reference	Reference	Reference
		Yes	9.56	19.66	1.10	0.273	(0.17,541.20)
	No Insurance Coverage	No	Reference	Reference	Reference	Reference	Reference
		Yes	338.59	1011.08	1.95	**0.05**	(0.96,118863.9)

Outcome	Interactions (SCD*SES)	Factor Levels	Odds Ratio	Linearized Std. Err.	t	P>|t|	[95%Conf. Interval]
**Learning Disability**	Gender	Female	Reference	Reference	Reference	Reference	Reference
		Male	1.08	0.81	0.10	0.921	(0.25,4.70)
	Age	<3	Reference	Reference	Reference	Reference	Reference
		3-5	-	-	-	-	-
		6-10	0.22	0.22	-1.52	0.13	(0.03,1.56)
		11-17	0.15	0.11	-2.65	**0.008**	(0.04,0.61)
	Maternal Education	< High school	Reference	Reference	Reference	Reference	Reference
		High school/GED	2.22	1.73	1.02	0.309	(0.48,10.26)
		> High school	7.84	7.34	2.20	**0.028**	(1.24,49.28)
	Region	Northeast	Reference	Reference	Reference	Reference	Reference
		Midwest	4.71	6.50	1.12	0.262	(0.31,70.66)
		South	1.89	2.84	0.43	0.67	(1.10,35.82)
		West	7.34	14.00	1.05	0.296	(0.17,309.54)
	Household Income (% federal poverty level)	<100	Reference	Reference	Reference	Reference	Reference
		100 to < 200	1.04	1.77	0.02	0.983	(0.04,29.35)
		≥ 200	1.24	1.75	0.15	0.879	(0.08,19.80)
	Race	Other	Reference	Reference	Reference	Reference	Reference
		Black	0.30	0.32	-1.11	0.265	(0.04, 2.48)
	Public Insurance	No	Reference	Reference	Reference	Reference	Reference
		Yes	0.52	1.12	-0.3	0.762	(0.01,36.12)
	Private Insurance	No	Reference	Reference	Reference	Reference	Reference
		Yes	0.01	0.03	-1.97	**0.05**	(0.00,0.99)
	Other Insurance	No	Reference	Reference	Reference	Reference	Reference
		Yes	0.55	0.91	-0.36	0.72	(0.02,13.91)
		No	Reference	Reference	Reference	Reference	Reference

	No Insurance Coverage	Yes	0.01	0.03	-1.75	**0.08**	(0.00,1.72)
Outcome	Interactions (SCD*SES)	Factor Levels	Odds Ratio	Linearized Std. Err.	t	P>|t|	[95%Conf. Interval]
**Trouble Hearing**	Gender	Female	Reference	Reference	Reference	Reference	Reference
		Male	2.38	1.63	1.26	0.207	(0.62,9.16)
	Age	<3	Reference	Reference	Reference	Reference	Reference
		3-5	0.66	0.75	-0.36	0.717	(0.07,6.06)
		6-10	0.31	0.52	-0.70	0.484	(0.01,8.33)
		11-17	0.57	0.66	-0.49	0.627	(0.06,5.62)
	Maternal Education	< High school	Reference	Reference	Reference	Reference	Reference
		High school/GED	1.73	1.61	0.59	0.556	(0.28,10.79)
		> High school	0.72	0.78	-0.31	0.760	(0.08,6.13)
	Region	Northeast	Reference	Reference	Reference	Reference	Reference
		Midwest	19.65	53.24	1.10	0.272	(0.10,4009.54)
		South	19.37	48.08	1.19	0.233	(0.15,2526.26)
		West	70.30	186.79	1.60	0.110	(0.38,12936.25)
	Household Income (% federal poverty level)	<100	Reference	Reference	Reference	Reference	Reference
		100 to < 200	0.04	0.07	-1.88	**0.061**	(0.00,1.16)
		≥ 200	-	-	-	-	-
	Race	Other	Reference	Reference	Reference	Reference	Reference
		Black	0.24	0.24	-1.44	0.149	(0.03,1.68)
	Public Insurance	No	Reference	Reference	Reference	Reference	Reference
		Yes	0.16	0.60	-0.50	0.620	(0.00,208.32)
	Private Insurance	No	Reference	Reference	Reference	Reference	Reference
		Yes	1.31	4.70	0.08	0.939	(0.00,1457.07)
	Other Insurance	No	Reference	Reference	Reference	Reference	Reference
		Yes	6.81	19.14	0.68	0.495	(0.03,1695.21)
	No Insurance Coverage	No	Reference	Reference	Reference	Reference	Reference
		Yes	1.86	7.49	0.15	0.877	(0.00,5005.92)

Outcome	Interactions (SCD*SES)	Factor Levels	Odds Ratio	Linearized Std. Err.	t	P>|t|	[95%Conf. Interval]
**Trouble Seeing**	Gender	Female	Reference	Reference	Reference	Reference	Reference
		Male	2.49	2.44	0.93	0.352	(0.36,17.07)
	Age	<3	Reference	Reference	Reference	Reference	Reference
		3-5	0.01	0.02	-2.26	**0.024**	(0.00,0.56)
		6-10	0.02	0.03	-2.29	**0.022**	(0.00,0.56)
		11-17	-	-	-	-	-
	Maternal Education	< High school	Reference	Reference	Reference	Reference	Reference
		High school/GED	0.03	0.07	-1.61	0.107	(0.00,2.11)
		> High school	-	-	-	-	-
	Region	Northeast	Reference	Reference	Reference	Reference	Reference
		Midwest	<0.01	0.01	-3.09	**0.002**	(0.00,0.12)
		South	<0.01	<0.01	-2.20	**0.028**	(0.00,0.28)
		West	-	-	-	-	-
	Household Income (% federal poverty level)	<100	Reference	Reference	Reference	Reference	Reference
		100 to < 200	0.02	0.07	-1.11	0.268	(0.00,21.60)
		≥ 200	0.57	1.54	-0.21	0.835	(0.00,115.13)
	Race	Other	Reference	Reference	Reference	Reference	Reference
		Black	-	-	-	-	-
	Public Insurance	No	Reference	Reference	Reference	Reference	Reference
		Yes	<0.01	<0.01	-2.10	**0.036**	(0.00,0.63)
	Private Insurance	No	Reference	Reference	Reference	Reference	Reference
		Yes	<0.01	<0.01	-2.55	**0.011**	(0.00,0.12)
	Other Insurance	No	Reference	Reference	Reference	Reference	Reference
		Yes	0.11	0.21	-1.17	0.241	(0.00,4.34)
	No Insurance Coverage	No	Reference	Reference	Reference	Reference	Reference
		Yes	0.24	0.77	-0.45	0.656	(0.00,133.64)

Health Impact							
Outcome	Interactions (SCD*SES)	Factor Levels	Odds Ratio	Linearized Std. Err.	t	P>|t|	[95%Conf. Interval]
**Limited in ability to crawl, walk, run, or play**	Gender	Female	Reference	Reference	Reference	Reference	Reference
		Male	1.31	0.89	0.39	0.694	(0.34,4.94)
	Age	<3	Reference	Reference	Reference	Reference	Reference
		3-5	0.42	0.31	-1.18	0.239	(0.10,1.79)
		6-10	3.16	2.36	1.54	0.124	(0.73,13.70)
		11-17	-	-	-	-	-
	Maternal Education	< High school	Reference	Reference	Reference	Reference	Reference
		High school/GED	0.44	0.43	-0.84	0.399	(0.06,3.02)
		> High school	1.10	0.94	0.11	0.916	(0.20,5.94)
	Region	Northeast	Reference	Reference	Reference	Reference	Reference
		Midwest	0.18	0.19	-1.66	**0.098**	(0.02,1.37)
		South	0.28	0.25	-1.43	0.152	(0.05,1.60)
		West	2.19	2.80	0.62	0.539	(0.18,26.86)
	Household Income (% federal poverty level)	<100	Reference	Reference	Reference	Reference	Reference
		100 to < 200	0.42	0.28	-1.28	0.202	(0.11,1.59)
		≥ 200	0.49	0.51	-0.69	0.491	(0.06,3.79)
	Race	Other	Reference	Reference	Reference	Reference	Reference
		Black	1.95	1.76	0.74	0.460	(0.33,11.45)
	Public Insurance	No	Reference	Reference	Reference	Reference	Reference
		Yes	0.12	0.19	-1.34	0.181	(0.01,2.66)
	Private Insurance	No	Reference	Reference	Reference	Reference	Reference
		Yes	0.15	0.25	-1.13	0.258	(0.01,4.04)
	Other Insurance	No	Reference	Reference	Reference	Reference	Reference
		Yes	0.91	1.41	-0.06	0.953	(0.04,19.11)
	No Insurance Coverage	No	Reference	Reference	Reference	Reference	Reference
		Yes	2.28	3.84	0.49	0.624	(0.08,61.85)

Outcome	Interactions (SCD*SES)	Factor Levels	Odds Ratio	Linearized Std. Err.	t	P>|t|	[95%Conf. Interval]
**Took prescription medication for at least three months**	Gender	Female	Reference	Reference	Reference	Reference	Reference
		Male	2.95	0.14	-2.58	**0.010**	(0.12,0.75)
	Age	<3	Reference	Reference	Reference	Reference	Reference
		3-5	0.24	0.17	-2.06	**0.040**	(0.06,0.94)
		6-10	1.69	1.15	0.78	0.437	(0.45,6.39)
		11-17	0.13	0.08	-3.27	**0.001**	(0.04,0.44)
	Maternal Education	< High school	Reference	Reference	Reference	Reference	Reference
		High school/GED	0.42	0.30	-1.22	0.223	(0.11,1.69)
		> High school	1.13	0.70	0.19	0.846	(0.34,3.79)
	Region	Northeast	Reference	Reference	Reference	Reference	Reference
		Midwest	0.77	0.61	-0.33	0.744	(0.17,3.60)
		South	0.73	0.57	-0.40	0.688	(0.16,3.42)
		West	0.90	0.94	-0.10	0.923	(0.12,6.92)
	Household Income (% federal poverty level)	<100	Reference	Reference	Reference	Reference	Reference
		100 to < 200	0.88	0.53	-0.21	0.835	(0.27,2.89)
		≥ 200	0.64	0.45	-0.63	0.529	(0.16,2.53)
	Race	Other	Reference	Reference	Reference	Reference	Reference
		Black	2.91	1.68	1.85	**0.065**	(0.94,9.04)
	Public Insurance	No	Reference	Reference	Reference	Reference	Reference
		Yes	1.03	0.86	0.04	0.972	(0.20,5.35)
	Private Insurance	No	Reference	Reference	Reference	Reference	Reference
		Yes	1.56	1.51	0.46	0.643	(0.24,10.40)
	Other Insurance	No	Reference	Reference	Reference	Reference	Reference
		Yes	1.73	1.82	0.52	0.601	(0.22,13.62)
	No Insurance Coverage	No	Reference	Reference	Reference	Reference	Reference
		Yes	3.31	3.39	1.17	0.243	(0.44,24.71)

Miss school because of illness or injury							
Outcome	Interactions (SCD*SES)	Factor Levels	Odds Ratio	Linearized Std. Err.	t	P>|t|	[95%Conf. Interval]
**# of days school missed because of illness or injury: 0**	Gender	Female	Reference	Reference	Reference	Reference	Reference
		Male	4.92	4.71	1.66	**0.097**	(0.75,32.22)
	Age	<3	Reference	Reference	Reference	Reference	Reference
		3-5	-	-	-	-	-
		6-10	0.15	0.21	-1.36	0.174	(0.01,2.31)
		11-17	0.09	0.14	-1.63	0.103	(0.01,1.62)
	Maternal Education	< High school	Reference	Reference	Reference	Reference	Reference
		High school/GED	1.13	1.23	0.12	0.908	(0.14,9.52)
		> High school	0.08	0.12	-1.65	0.100	(0.00,1.64)
	Region	Northeast	Reference	Reference	Reference	Reference	Reference
		Midwest	0.76	0.70	-0.30	0.765	(0.12,4.70)
		South	0.85	0.95	-0.14	0.885	(0.09,7.65)
		West	-	-	-	-	-
	Household Income (% federal poverty level)	<100	Reference	Reference	Reference	Reference	Reference
		100 to < 200	1.05	1.16	0.04	0.967	(0.12,9.16)
		≥ 200	2.18	3.78	0.45	0.654	(0.07,65.65)
	Race	Other	Reference	Reference	Reference	Reference	Reference
		Black	4.48	5.19	1.30	0.196	(0.46,43.48)
	Public Insurance	No	Reference	Reference	Reference	Reference	Reference
		Yes	1.07	1.63	0.04	0.965	(0.05,21.23)
	Private Insurance	No	Reference	Reference	Reference	Reference	Reference
		Yes	10.02	18.76	1.23	0.219	(0.25,395.29)
	Other Insurance	No	Reference	Reference	Reference	Reference	Reference
		Yes	0.49	0.77	-0.46	0.649	(0.22,10.88)
	No Insurance Coverage	No	Reference	Reference	Reference	Reference	Reference
		Yes	-	-	-	-	-

Outcome	Interactions (SCD*SES)	Factor Levels	Odds Ratio	Linearized Std. Err.	t	P>|t|	[95%Conf. Interval]
**# of days school missed because of illness or injury: 1-10**	Gender	Female	Reference	Reference	Reference	Reference	Reference
		Male	0.35	0.24	-1.54	0.124	(0.09,1.33)
	Age	<3	Reference	Reference	Reference	Reference	Reference
		3-5	-	-	-	-	-
		6-10	3.06	2.58	1.32	0.186	(0.58,16.05)
		11-17	7.56	7.73	1.98	**0.048**	(1.02,56.17)
	Maternal Education	< High school	Reference	Reference	Reference	Reference	Reference
		High school/GED	3.31	3.20	1.23	0.218	(0.49,22.15)
		> High school	3.35	3.10	1.30	0.192	(0.54,20.59)
	Region	Northeast	Reference	Reference	Reference	Reference	Reference
		Midwest	4.02	3.76	1.49	0.137	(0.64,25.22)
		South	3.36	3.28	1.24	0.216	(0.49,22.91)
		West	14.18	23.40	1.61	0.108	(0.56,361.74)
	Household Income (% federal poverty level)	<100	Reference	Reference	Reference	Reference	Reference
		100 to < 200	0.37	0.27	-1.34	0.181	(0.09,1.59)
		≥ 200	0.30	0.36	-1.01	0.315	(0.03,3.13)
	Race	Other	Reference	Reference	Reference	Reference	Reference
		Black	0.15	0.15	-1.86	**0.063**	(0.02,1.11)
	Public Insurance	No	Reference	Reference	Reference	Reference	Reference
		Yes	0.49	0.63	-0.56	0.577	(0.04,6.19)
	Private Insurance	No	Reference	Reference	Reference	Reference	Reference
		Yes	0.42	0.67	-0.54	0.587	(0.02,9.50)
	Other Insurance	No	Reference	Reference	Reference	Reference	Reference
		Yes	0.50	0.78	-0.44	0.657	(0.02,10.63)
	No Insurance Coverage	No	Reference	Reference	Reference	Reference	Reference
		Yes	0.99	1.62	-0.01	0.994	(0.04,24.63)

Outcome	Interactions (SCD*SES)	Factor Levels	Odds Ratio	Linearized Std. Err.	t	P>|t|	[95%Conf. Interval]
**# of days school missed because of illness or injury: 11-20**	Gender	Female	Reference	Reference	Reference	Reference	Reference
		Male	1.92	1.66	0.76	0.449	(0.35,10.43)
	Age	<3	Reference	Reference	Reference	Reference	Reference
		3-5	-	-	-	-	-
		6-10	0.23	0.22	-1.55	0.121	(0.04,1.47)
		11-17	0.03	0.05	-2.53	**0.012**	(0.00,0.47)
	Maternal Education	< High school	Reference	Reference	Reference	Reference	Reference
		High school/GED	0.03	0.09	-1.29	0.199	(0.00,6.11)
		> High school	0.14	0.19	-1.45	0.148	(0.01,2.01)
	Region	Northeast	Reference	Reference	Reference	Reference	Reference
		Midwest	1.04	1.68	0.03	0.978	(0.04,24.34)
		South	1.21	2.12	0.11	0.915	(0.04,37.98)
		West	-	-	-	-	-
	Household Income (% federal poverty level)	<100	Reference	Reference	Reference	Reference	Reference
		100 to < 200	3.38	5.23	0.79	0.431	(0.16,70.30)
		≥ 200	1.57	2.49	0.29	0.774	(0.07,34.95)
	Race	Other	Reference	Reference	Reference	Reference	Reference
		Black	5.33	12.34	0.72	0.470	(0.06,502.38)
	Public Insurance	No	Reference	Reference	Reference	Reference	Reference
		Yes	2.20	3.50	0.49	0.621	(0.10,49.97)
	Private Insurance	No	Reference	Reference	Reference	Reference	Reference
		Yes	7.61	13.95	1.11	0.268	(0.21,277.71)
	Other Insurance	No	Reference	Reference	Reference	Reference	Reference
		Yes	-	-	-	-	-
	No Insurance Coverage	No	Reference	Reference	Reference	Reference	Reference
		Yes	22.14	49.61	1.38	0.167	(0.27,1799.65)

Outcome	Interactions (SCD*SES)	Factor Levels	Odds Ratio	Linearized Std. Err.	t	P>|t|	[95%Conf. Interval]
**# of days school missed because of illness or injury: 21-30**	Gender	Female	Reference	Reference	Reference	Reference	Reference
		Male	0.97	2.13	-0.01	0.989	(0.01,71.64)
	Age	<3	Reference	Reference	Reference	Reference	Reference
		3-5	-	-	-	-	-
		6-10	1.95	1.54	0.85	0.397	(0.41,9.17)
		11-17	-	-	-	-	-
	Maternal Education	< High school	Reference	Reference	Reference	Reference	Reference
		High school/GED	0.83	0.89	-0.18	0.860	(0.10,6.74)
		> High school	0.15	0.29	-1.01	0.312	(0.00,5.77)
	Region	Northeast	Reference	Reference	Reference	Reference	Reference
		Midwest	-	-	-	-	-
		South	0.28	0.28	-1.25	0.210	(0.04,2.05)
		West	-	-	-	-	-
	Household Income (% federal poverty level)	<100	Reference	Reference	Reference	Reference	Reference
		100 to < 200	0.57	0.95	-0.34	0.735	(0.02,14.97)
		≥ 200	26.71	54.94	1.60	0.111	(0.47,1513.12)
	Race	Other	Reference	Reference	Reference	Reference	Reference
		Black	-	-	-	-	-
	Public Insurance	No	Reference	Reference	Reference	Reference	Reference
		Yes	<0.01	0.02	-1.48	0.138	(0.00,5.81)
	Private Insurance	No	Reference	Reference	Reference	Reference	Reference
		Yes	0.02	0.05	-1.73	**0.085**	(0.00,1.71)
	Other Insurance	No	Reference	Reference	Reference	Reference	Reference
		Yes	0.56	1.10	-0.30	0.767	(0.01,26.41)
	No Insurance Coverage	No	Reference	Reference	Reference	Reference	Reference
		Yes	-	-	-	-	-
**Healthcare and special education services use (past 12 months)**							
Outcome	Interactions (SCD*SES)	Factor Levels	Odds Ratio	Linearized Std. Err.	t	P>|t|	[95%Conf. Interval]

**Saw a medical specialist**	Gender	Female	Reference	Reference	Reference	Reference	Reference
		Male	0.73	0.37	-0.62	0.538	(0.27,1.97)
	Age	<3	Reference	Reference	Reference	Reference	Reference
		3-5	2.08	1.95	0.78	0.436	(0.33,13.11)
		6-10	7.85	6.19	2.61	**0.009**	(1.67,36.94)
		11-17	1.74	1.47	0.66	0.508	(0.33,9.09)
	Maternal Education	< High school	Reference	Reference	Reference	Reference	Reference
		High school/GED	0.73	0.60	-0.38	0.704	(0.15,3.61)
		> High school	1.59	1.19	0.62	0.535	(0.37,6.91)
	Region	Northeast	Reference	Reference	Reference	Reference	Reference
		Midwest	0.27	0.24	-1.48	0.139	(0.05,1.53)
		South	0.68	0.54	-0.49	0.627	(0.14,3.23)
		West	0.06	0.09	-1.94	**0.053**	(0.00,1.04)
	Household Income (% federal poverty level)	<100	Reference	Reference	Reference	Reference	Reference
		100 to < 200	0.55	0.37	-0.90	0.369	(0.15,2.05)
		≥ 200	0.53	0.39	-0.87	0.385	(0.13,2.20)
	Race	Other	Reference	Reference	Reference	Reference	Reference
		Black	1.93	1.12	1.13	0.258	(0.62,6.02)
	Public Insurance	No	Reference	Reference	Reference	Reference	Reference
		Yes	0.98	0.90	-0.02	0.981	(0.16,5.91)
	Private Insurance	No	Reference	Reference	Reference	Reference	Reference
		Yes	1.83	1.82	0.61	0.544	(0.26,12.95)
	Other Insurance	No	Reference	Reference	Reference	Reference	Reference
		Yes	2.06	2.46	0.61	0.544	(0.20,21.47)
	No Insurance Coverage	No	Reference	Reference	Reference	Reference	Reference
		Yes	0.81	0.97	-0.17	0.862	(0.08,8.52)
Outcome	Interactions (SCD*SES)	Factor Levels	Odds Ratio	Linearized Std. Err.	t	P>|t|	[95%Conf. Interval]
**Saw a therapist**	Gender	Female	Reference	Reference	Reference	Reference	Reference
		Male	2.59	1.80	1.37	0.170	(0.67,10.12)
	Age	<3	Reference	Reference	Reference	Reference	Reference

		3-5	4.54	5.91	1.16	0.246	(0.35,58.41)
		6-10	4.27	6.42	0.97	0.334	(0.22,81.43)
		11-17	1.42	2.07	0.24	0.812	(0.08,25.06)
	Maternal Education	< High school	Reference	Reference	Reference	Reference	Reference
		High school/GED	0.72	0.51	-0.46	0.644	(0.18,2.93)
		> High school	0.95	0.75	-0.07	0.944	(0.20,4.45)
	Region	Northeast	Reference	Reference	Reference	Reference	Reference
		Midwest	3.56	5.28	0.86	0.391	(0.19,65.24)
		South	1.43	2.24	0.23	0.821	(0.07,31.00)
		West	3.52	7.01	0.63	0.527	(0.07,174.78)
	Household Income (% federal poverty level)	<100	Reference	Reference	Reference	Reference	Reference
		100 to < 200	0.47	0.71	-0.50	0.615	(0.02,9.16)
		≥ 200	0.03	0.06	-1.75	**0.081**	(0.00,1.55)
	Race	Other	Reference	Reference	Reference	Reference	Reference
		Black	0.95	1.14	-0.04	0.966	(0.09,10.01)
	Public Insurance	No	Reference	Reference	Reference	Reference	Reference
		Yes	2.14	2.84	0.57	0.568	(0.16,29.02)
	Private Insurance	No	Reference	Reference	Reference	Reference	Reference
		Yes	3.36	4.42	0.92	0.356	(0.25,44.40)
	Other Insurance	No	Reference	Reference	Reference	Reference	Reference
		Yes	1.43	2.31	0.22	0.822	(0.06,33.61)
	No Insurance Coverage	No	Reference	Reference	Reference	Reference	Reference
		Yes	19.06	25.23	2.23	**0.026**	(1.42,256.05)
Outcome	Interactions (SCD*SES)	Factor Levels	Odds Ratio	Linearized Std. Err.	t	P>|t|	[95%Conf. Interval]
**Saw an optometrist. ophthalmologist, or eye doctor**	Gender	Female	Reference	Reference	Reference	Reference	Reference
		Male	0.70	0.37	-0.67	0.501	(0.25,1.97)
	Age	<3	Reference	Reference	Reference	Reference	Reference
		3-5	0.48	0.56	-0.63	0.531	(0.05,4.68)
		6-10	0.75	0.82	-0.26	0.795	(0.09,6.45)
		11-17	0.42	0.43	-0.85	0.394	(0.06,3.09)

	Maternal Education	< High school	Reference	Reference	Reference	Reference	Reference
		High school/GED	0.53	0.44	-0.77	0.444	(0.10,2.71)
		> High school	2.80	2.08	1.38	0.167	(0.65,12.07)
	Region	Northeast	Reference	Reference	Reference	Reference	Reference
		Midwest	0.60	0.47	-0.66	0.512	(0.13,2.80)
		South	1.50	1.20	0.51	0.611	(0.31,7.18)
		West	0.89	0.91	-0.12	0.906	(0.12,6.60)
	Household Income (% federal poverty level)	<100	Reference	Reference	Reference	Reference	Reference
		100 to < 200	0.27	0.17	-2.05	**0.041**	(0.08,0.95)
		≥ 200	0.73	0.61	-0.37	0.709	(0.14,3.75)
	Race	Other	Reference	Reference	Reference	Reference	Reference
		Black	1.51	1.06	0.58	0.560	(0.38,6.02)
	Public Insurance	No	Reference	Reference	Reference	Reference	Reference
		Yes	0.69	0.72	-0.35	0.725	(0.09,5.36)
	Private Insurance	No	Reference	Reference	Reference	Reference	Reference
		Yes	1.82	2.19	0.5	0.617	(0.17,19.18)
	Other Insurance	No	Reference	Reference	Reference	Reference	Reference
		Yes	1.68	1.87	0.47	0.639	(0.19,14.93)
	No Insurance Coverage	No	Reference	Reference	Reference	Reference	Reference
		Yes	0.16	0.27	-1.11	0.269	(0.00,4.07)
**Multiple visits to emergency room**							
Outcome	Interactions (SCD*SES)	Factor Levels	Odds Ratio	Linearized Std. Err.	t	P>|t|	[95%Conf. Interval]
**Had surgery or another surgical procedure**	Gender	Female	Reference	Reference	Reference	Reference	Reference
		Male	15.47	16.92	2.51	**0.012**	(1.81,132.27)
	Age	<3	Reference	Reference	Reference	Reference	Reference
		3-5	1.55	1.68	0.40	0.689	(0.18,13.10)
		6-10	4.29	4.84	1.29	0.196	(0.47,39.24)
		11-17	1.41	1.65	0.30	0.767	(0.14,13.97)
		< High school	Reference	Reference	Reference	Reference	Reference

	Maternal Education	High school/GED	6.68	13.58	0.93	0.351	(0.12,361.89)
		> High school	0.45	0.78	-0.46	0.645	(0.01,13.94)
	Region	Northeast	Reference	Reference	Reference	Reference	Reference
		Midwest	0.86	0.72	-0.18	0.861	(0.17,4.46)
		South	-	-	-	-	-
		West	-	-	-	-	-
	Household Income (% federal poverty level)	<100	Reference	Reference	Reference	Reference	Reference
		100 to < 200	0.09	0.11	-1.87	**0.061**	(0.00,1.12)
		≥ 200	21.22	28.16	2.30	**0.022**	(1.57,287.05)
	Race	Other	Reference	Reference	Reference	Reference	Reference
		Black	0.31	0.32	-1.13	0.258	(0.04,2.34)
	Public Insurance	No	Reference	Reference	Reference	Reference	Reference
		Yes	<0.01	<0.01	-3.18	**0.002**	(0.00,0.13)
	Private Insurance	No	Reference	Reference	Reference	Reference	Reference
		Yes	<0.01	0.01	-3.27	**0.001**	(0.00,0.10)
	Other Insurance	No	Reference	Reference	Reference	Reference	Reference
		Yes	0.34	0.80	-0.46	0.645	(0.00,32.74)
	No Insurance Coverage	No	Reference	Reference	Reference	Reference	Reference
		Yes	0.11	0.15	-1.65	0.100	(0.00,1.52)
Outcome	Interactions (SCD*SES)	Factor Levels	Odds Ratio	Linearized Std. Err.	t	P>|t|	[95%Conf. Interval]
**# of Visits to emergency room:none**	Gender	Female	Reference	Reference	Reference	Reference	Reference
		Male	1.03	0.50	0.05	0.957	(0.39,2.68)
	Age	<3	Reference	Reference	Reference	Reference	Reference
		3-5	0.99	0.70	-0.02	0.985	(0.24,3.98)
		6-10	1.27	0.91	0.34	0.734	(0.32,5.14)
		11-17	5.00	3.62	2.23	**0.026**	(1.21,20.69)
	Maternal Education	< High school	Reference	Reference	Reference	Reference	Reference
		High school/GED	4.33	2.84	2.23	**0.026**	(1.19,15.70)
		> High school	2.34	1.41	1.42	0.157	(0.72,7.64)

	Region	Northeast	Reference	Reference	Reference	Reference	Reference
		Midwest	1.10	0.83	0.13	0.896	(0.25,4.81)
		South	0.84	0.57	-0.25	0.801	(0.23,3.16)
		West	1.56	1.72	0.41	0.684	(0.18,13.55)
	Household Income (% federal poverty level)	<100	Reference	Reference	Reference	Reference	Reference
		100 to < 200	0.47	0.26	-1.38	0.169	(0.16,1.38)
		≥ 200	0.59	0.41	-0.76	0.447	(0.15,2.29)
	Race	Other	Reference	Reference	Reference	Reference	Reference
		Black	0.45	0.31	-1.16	0.245	(0.11,1.74)
	Public Insurance	No	Reference	Reference	Reference	Reference	Reference
		Yes	0.33	0.35	-1.05	0.293	(0.04,2.60)
	Private Insurance	No	Reference	Reference	Reference	Reference	Reference
		Yes	0.22	0.24	-1.40	0.161	(0.03,1.83)
	Other Insurance	No	Reference	Reference	Reference	Reference	Reference
		Yes	0.87	1.03	-0.12	0.907	(0.09.8.83)
	No Insurance Coverage	No	Reference	Reference	Reference	Reference	Reference
		Yes	0.13	0.16	-1.68	**0.094**	(0.02,1.41)
Outcome	Interactions (SCD*SES)	Factor Levels	Odds Ratio	Linearized Std. Err.	t	P>|t|	[95%Conf. Interval]
**# of Visits to emergency room: 1**	Gender	Female	Reference	Reference	Reference	Reference	Reference
		Male	2.82	1.87	1.56	0.119	(0.77,10.35)
	Age	<3	Reference	Reference	Reference	Reference	Reference
		3-5	0.63	0.53	-0.55	0.584	(0.12,3.23)
		6-10	0.49	0.38	-0.91	0.365	(0.11,2.27)
		11-17	0.51	0.34	-1.00	0.318	(0.14,1.91)
	Maternal Education	< High school	Reference	Reference	Reference	Reference	Reference
		High school/GED	0.16	0.11	-2.58	**0.010**	(0.04,0.65)
		> High school	0.16	0.10	-2.97	**0.003**	(0.05,0.53)
	Region	Northeast	Reference	Reference	Reference	Reference	Reference
		Midwest	0.05	0.06	-2.46	**0.014**	(0.01,0.55)

		South	0.18	0.16	-1.89	**0.059**	(0.03,1.07)
		West	0.02	0.04	-2.03	**0.043**	(0.00,0.88)
	Household Income (% federal poverty level)	<100	Reference	Reference	Reference	Reference	Reference
		100 to < 200	4.69	3.64	1.99	**0.047**	(1.02,21.54)
		≥ 200	4.57	4.34	1.60	0.110	(0.71,29.54)
	Race	Other	Reference	Reference	Reference	Reference	Reference
		Black	2.20	1.96	0.89	0.375	(0.38,12.61)
	Public Insurance	No	Reference	Reference	Reference	Reference	Reference
		Yes	7.90	11.85	1.38	0.169	(0.42,150.18)
	Private Insurance	No	Reference	Reference	Reference	Reference	Reference
		Yes	1.52	2.43	0.26	0.795	(0.06,35.18)
	Other Insurance	No	Reference	Reference	Reference	Reference	Reference
		Yes	0.67	1.15	-0.23	0.817	(0.023,19.55)
	No Insurance Coverage	No	Reference	Reference	Reference	Reference	Reference
		Yes	4.62	8.12	0.87	0.385	(0.15,145.95)
Outcome	Interactions (SCD*SES)	Factor Levels	Odds Ratio	Linearized Std. Err.	t	P>|t|	[95%Conf. Interval]
**# of Visits to emergency room: 2-3**	Gender	Female	Reference	Reference	Reference	Reference	Reference
		Male	0.72	0.45	-0.52	0.603	(0.21,2.45)
	Age	<3	Reference	Reference	Reference	Reference	Reference
		3-5	1.84	1.63	0.69	0.490	(0.32,10.48)
		6-10	0.77	0.69	-0.29	0.770	(0.13,4.43)
		11-17	0.45	0.42	-0.85	0.395	(0.07,2.84)
	Maternal Education	< High school	Reference	Reference	Reference	Reference	Reference
		High school/GED	0.12	0.12	-2.09	**0.037**	(0.16,0.88)
		> High school	0.81	0.55	-0.30	0.761	(0.21,3.09)
	Region	Northeast	Reference	Reference	Reference	Reference	Reference
		Midwest	4.67	5.49	1.31	0.191	(0.46,46.95)
		South	2.47	2.70	0.83	0.410	(0.29,21.13)
		West	6.42	10.10	1.18	0.237	(0.29,140.52)

	Household Income (% federal poverty level)	<100	Reference	Reference	Reference	Reference	Reference
		100 to < 200	0.40	0.37	-1.00	0.318	(0.07,2.40)
		≥ 200	1.15	0.96	0.17	0.863	(0.23,5.91)
	Race	Other	Reference	Reference	Reference	Reference	Reference
		Black	2.00	1.58	0.88	0.380	(0.43,9.39)
	Public Insurance	No	Reference	Reference	Reference	Reference	Reference
		Yes	0.92	0.99	-0.08	0.936	(0.11,7.60)
	Private Insurance	No	Reference	Reference	Reference	Reference	Reference
		Yes	2.68	3.42	0.78	0.437	(0.22,32.54)
	Other Insurance	No	Reference	Reference	Reference	Reference	Reference
		Yes	1.90	2.53	0.48	0.632	(0.14,26.00)
	No Insurance Coverage	No	Reference	Reference	Reference	Reference	Reference
		Yes	0.74	0.98	-0.23	0.821	(0.06,9.77)
Outcome	Interactions (SCD*SES)	Factor Levels	Odds Ratio	Linearized Std. Err.	t	P>|t|	[95%Conf. Interval]
**# of Visits to emergency room: 4-5**	Gender	Female	Reference	Reference	Reference	Reference	Reference
		Male	0.68	0.47	-0.56	0.576	(0.18,2.62)
	Age	<3	Reference	Reference	Reference	Reference	Reference
		3-5	1.67	1.88	0.46	0.648	(0.18,15.21)
		6-10	0.25	0.43	-0.81	0.417	(0.01,7.15)
		11-17	0.08	0.11	-1.74	**0.083**	(0.00,1.40)
	Maternal Education	< High school	Reference	Reference	Reference	Reference	Reference
		High school/GED	12.04	16.67	1.80	**0.073**	(0.80,182.30)
		> High school	1.84	2.56	0.44	0.662	(0.12,28.38)
	Region	Northeast	Reference	Reference	Reference	Reference	Reference
		Midwest	30.21	55.77	1.85	**0.065**	(0.81,1131.97)
		South	7.38	11.47	1.29	0.199	(0.35,155.83)
		West	-	-	-	-	-
	Household Income (% federal	<100	Reference	Reference	Reference	Reference	Reference
		100 to < 200	10.23	12.70	1.87	**0.062**	(0.89,117.08)

	poverty level)	≥ 200	1.21	3.45	0.07	0.947	(0.00,327.10)
	Race	Other	Reference	Reference	Reference	Reference	Reference
		Black	0.63	0.81	-0.36	0.720	(0.05,7.73)
	Public Insurance	No	Reference	Reference	Reference	Reference	Reference
		Yes	77.03	193.29	1.73	**0.084**	(0.56,10606.64)
	Private Insurance	No	Reference	Reference	Reference	Reference	Reference
		Yes	57.30	114.78	2.02	**0.044**	(1.12,2921.31)
	Other Insurance	No	Reference	Reference	Reference	Reference	Reference
		Yes	-	-	-	-	-
	No Insurance Coverage	No	Reference	Reference	Reference	Reference	Reference
		Yes	439.96	1112.70	2.41	**0.016**	(3.07,62980.07)
Outcome	Interactions (SCD*SES)	Factor Levels	Odds Ratio	Linearized Std. Err.	t	P>|t|	[95%Conf. Interval]
**# of Visits to emergency room: 6-16+**	Gender	Female	Reference	Reference	Reference	Reference	Reference
		Male	0.18	0.21	-1.47	0.141	(0.02,1.77)
	Age	<3	Reference	Reference	Reference	Reference	Reference
		3-5	0.22	0.44	-0.76	0.446	(0.00,11.09)
		6-10	10.62	10.18	2.47	**0.014**	(1.62,69.66)
		11-17	0.16	0.29	-1.02	0.310	(0.00,5.51)
	Maternal Education	< High school	Reference	Reference	Reference	Reference	Reference
		High school/GED	0.67	0.71	-0.38	0.707	(0.09,5.28)
		> High school	-	-	-	-	-
	Region	Northeast	Reference	Reference	Reference	Reference	Reference
		Midwest	0.01	0.02	-2.03	**0.043**	(0.00,0.85)
		South	-	-	-	-	-
		West	-	-	-	-	-
	Household Income (% federal poverty level)	<100	Reference	Reference	Reference	Reference	Reference
		100 to < 200	0.70	0.75	-0.33	0.741	(0.09,5.69)
		≥ 200	0.59	0.63	-0.49	0.625	(0.07,4.85)
	Race	Other	Reference	Reference	Reference	Reference	Reference

		Black	2.24	1.84	0.98	0.329	(0.44,11.29)
	Public Insurance	No	Reference	Reference	Reference	Reference	Reference
		Yes	11.74	25.02	1.16	0.248	(0.18,768.57)
	Private Insurance	No	Reference	Reference	Reference	Reference	Reference
		Yes	432.79	939.16	2.80	**0.005**	(6.12,30616.45)
	Other Insurance	No	Reference	Reference	Reference	Reference	Reference
		Yes	96.18	243.07	1.81	**0.071**	(0.67,13720.4)
	No Insurance Coverage	No	Reference	Reference	Reference	Reference	Reference
		Yes	172.02	423.53	2.09	**0.037**	(1.37,21587.03)
**Healthcare Barrier**							
Outcome	Interactions (SCD*SES)	Factor Levels	Odds Ratio	Linearized Std. Err.	t	P>|t|	[95%Conf. Interval]
**Couldn't get an appointment**	Gender	Female	Reference	Reference	Reference	Reference	Reference
		Male	0.15	0.14	-1.96	**0.050**	(0.21,1.00)
	Age	<3	Reference	Reference	Reference	Reference	Reference
		3-5	0.82	0.97	-0.17	0.867	(0.08,8.45)
		6-10	2.30	2.05	0.93	0.353	(0.40,13.30)
		11-17	-	-	-	-	-
	Maternal Education	< High school	Reference	Reference	Reference	Reference	Reference
		High school/GED	9.20	13.54	1.51	0.132	(0.51,165.37)
		> High school	35.09	58.08	2.15	**0.032**	(1.36,903.83)
	Region	Northeast	Reference	Reference	Reference	Reference	Reference
		Midwest	3.20	5.80	0.64	0.522	(0.09,112.47)
		South	23.62	46.80	1.60	0.111	(0.48,1153.36)
		West	6.75	18.88	0.68	0.495	(0.03,1638.93)
	Household Income (% federal poverty level)	<100	Reference	Reference	Reference	Reference	Reference
		100 to < 200	4.53	4.26	1.61	0.108	(0.72,28.71)
		≥ 200	-	-	-	-	-
	Race	Other	Reference	Reference	Reference	Reference	Reference
		Black	0.19	0.25	-1.26	0.208	(0.01,2.50)
	Public	No	Reference	Reference	Reference	Reference	Reference

	Insurance	Yes	1.32	1.72	0.21	0.831	(0.11,16.94)
	Private Insurance	No	Reference	Reference	Reference	Reference	Reference
		Yes	0.37	0.52	-0.71	0.478	(0.02,5.67)
	Other Insurance	No	Reference	Reference	Reference	Reference	Reference
		Yes	10.58	12.34	2.02	**0.043**	(1.07,104.42)
	No Insurance Coverage	No	Reference	Reference	Reference	Reference	Reference
		Yes	20.21	29.67	2.05	**0.041**	(1.13,360.80)
Outcome	Interactions (SCD*SES)	Factor Levels	Odds Ratio	Linearized Std. Err.	t	P>|t|	[95%Conf. Interval]
**No transportation**	Gender	Female	Reference	Reference	Reference	Reference	Reference
		Male	5.41	4.54	2.01	**0.045**	(1.04,20.08)
	Age	<3	Reference	Reference	Reference	Reference	Reference
		3-5	0.41	0.48	-0.76	0.446	(0.04,3.98)
		6-10	0.79	0.92	-0.20	0.839	(0.08,7.81)
		11-17	-	-	-	-	-
	Maternal Education	< High school	Reference	Reference	Reference	Reference	Reference
		High school/GED	1.07	1.21	0.06	0.952	(0.12,9.96)
		> High school	-	-	-	-	-
	Region	Northeast	Reference	Reference	Reference	Reference	Reference
		Midwest	0.41	0.45	-0.81	0.419	(0.05,3.60)
		South	0.12	0.19	-1.36	0.173	(0.01,2.55)
		West	-	-	-	-	-
	Household Income (% federal poverty level)	<100	Reference	Reference	Reference	Reference	Reference
		100 to < 200	0.79	0.96	-0.19	0.848	(0.08,8.41)
		≥ 200	9.80	12.60	1.78	**0.076**	(0.79,122.14)
	Race	Other	Reference	Reference	Reference	Reference	Reference
		Black	0.09	0.09	-2.42	**0.016**	(0.01,0.63)
	Public Insurance	No	Reference	Reference	Reference	Reference	Reference
		Yes	0.03	0.04	-2.82	**0.005**	(0.00,0.35)
	Private Insurance	No	Reference	Reference	Reference	Reference	Reference
		Yes	0.03	0.04	-2.18	**0.030**	(0.00,0.68)

	Other Insurance	No	Reference	Reference	Reference	Reference	Reference
		Yes	0.42	0.67	-0.54	0.588	(0.02,9.92)
	No Insurance Coverage	No	Reference	Reference	Reference	Reference	Reference
		Yes	0.31	0.37	-0.98	0.326	(0.03,3.21)
